# Long-term trends of carbon monoxide over the North Pacific upper troposphere from in-situ airplane measurements 1991–2018

**DOI:** 10.1371/journal.pone.0359855

**Published:** 2026-10-13

**Authors:** Kuo-Ying Wang, Philippe Nedelec, Valerie Thouret, Hannah Clark, Andreas Wahner, Andreas Petzold

**Affiliations:** 1 Department of Atmospheric Sciences, National Central University, Chung-Li, Taiwan; 2 Laboratoire d’Aerologie, Centre National de la Recherche Scientifique, Observatoire Midi-Pyrenees, Toulouse, France; 3 Forschungszentrum Julich GmbH, Institut fur Energie und Klimaforschung, IEK-8 Troposphare, Julich, Germany; Guizhou University of Finance and Economics, CHINA

## Abstract

Carbon monoxide (CO) is not a greenhouse gas, but as the dominant sink of the hydroxyl radical it governs the oxidising capacity of the troposphere and so indirectly forces climate. Its trends in the North Pacific upper troposphere matter disproportionately: this is the principal outflow corridor for Asian anthropogenic emissions, the fastest-growing combustion source of the past half-century, yet it has almost no routine ground-based monitoring and satellite retrievals resolve it poorly. We therefore combine in-situ CO measurements from the In-service Aircraft for a Global Observing System (IAGOS; 2012–2018) with earlier in-situ data from NASA/NSF/NOAA campaigns (1991–2019), the Measurements of Pollution in the Troposphere (MOPITT) satellite record, and the Integrated Modelling System (IMS) global chemistry transport model. Trends are computed separately for the monthly maximum, percentile, minimum and mean statistics, using a 10,000-sample perturbation regression framework. Mean CO increases by 0.15 ppb *month*^–1^ (18.0 ppb *decade*^–1^; 95% CI: −0.02 to 0.32) over $150^{\circ }E-140^{\circ }W$, $45^{\circ }N-60^{\circ }N$ and by 0.06 ppb *month*^–1^ (7.2 ppb *decade*^–1^; 95% CI: −0.04 to 0.16) over $25^{\circ }N-45^{\circ }N$; both intervals include zero but lie predominantly positive. The monthly maximum rises significantly over $25^{\circ }N-45^{\circ }N$, by 0.31 ppb *month*^–1^ (37.2 ppb *decade*^–1^; 95% CI: 0.06 to 0.57), while the monthly minimum declines weakly. The most polluted air masses over the North Pacific are thus becoming more polluted even as background CO falls. The North Atlantic upper troposphere shows significantly decreasing CO over the same period. The opposing signs track the opposing trajectories of the upwind inventories: Asian emissions rose through most of the record and are lifted efficiently by summertime convection and warm conveyor belts, whereas North American and European emissions fell steadily and the Atlantic receives a larger share of CO-poor stratospheric air.

## Introduction

Given the significant scale of anthropogenic emissions originating from Asian regions since the 1970s [1], there is a critical need for routine measurements of anthropogenic air pollutants in the Pacific upper troposphere. Such measurements would not only enhance our understanding of the spatial and temporal extent of anthropogenic emissions in this region but also facilitate the development and validation of models aimed at predicting the export of air pollution from Asian sources [2].

The impacts of atmospheric short-lived species such as carbon monoxide (CO) and nitrogen oxides (NOx) are challenging to track compared to longer-lived species like carbon dioxide (*CO*_2_), methane (*CH*_4_), and chlorofluorocarbons (CFCs) [3]. This difficulty arises from the faster chemistry involved, compounded by the complex interactions of these short-lived species with various Earth system components such as the lithosphere, biosphere, hydrosphere, and cryosphere, particularly within the troposphere. Moreover, the tropospheric dynamics, including temperature, wind patterns, humidity, and observational constraints, pose additional challenges in tracking the long-term trends of these short-lived species originating from anthropogenic emissions.

In the lower troposphere, elevated temperatures accelerate chemical reaction rates, while abundant gas-phase water vapor facilitates the production of hydroxyl radicals (OH), leading to shorter chemical lifetimes for short-lived species like CO compared to the upper troposphere, where lower temperatures and drier air prevail [3,4]. Because OH is the dominant sink of CO, spatial and seasonal structure in OH translates directly into structure in the CO lifetime, and that structure is now known to be substantial. Kuttippurath et al. [5] document a seasonal OH minimum over the Indian Ocean, most pronounced in spring with a secondary minimum in autumn, arising from the coupled chemistry of *SO*_2_, CO, *O*_3_ and water vapour in that region. Their result is a caution rather than a direct comparison for the present study: it concerns a different ocean basin, and no equivalent characterisation of the seasonal OH field is currently available for the North Pacific. Its significance here is methodological. It demonstrates that basin-scale regions of depressed oxidising capacity exist and are seasonally organised, so that a single globally averaged CO lifetime is not an adequate basis for interpreting regional CO records. Where such a minimum is present, CO enhancements survive further downwind than a uniform-OH assumption would predict. Establishing whether a comparable seasonal structure exists over the North Pacific would materially improve the attribution of the trends we report, and we identify it as a priority for future work. The study by Park et al. [6] offers critical insights into the fate of pollution from the 2015 Indonesian fire season, revealing the extensive transport and chemical evolution of pollutants into the UTLS. The findings underscore the importance of integrated observational and modeling approaches for understanding and mitigating the atmospheric impacts of large-scale fire events, with implications for global climate and air quality management. Froidevaux et al. [7] studied the tropical upper-tropospheric trends in ozone and CO. Gaudel et al. [8] studied tropical tropospheric ozone distribution and trends from in situ and satellite data. During August 25 to September 6, 2004, observations from the EOS Microwave Limb Sounder (MLS) showed elevated carbon monoxide (CO) and dense high clouds over the Tibetan Plateau and the Bay of Bengal, indicating significant convective outflow of South Asian pollution. A global 3-D chemical transport model was used to analyze these observations, confirming that the pollution primarily originates from anthropogenic sources in South Asia, with a small contribution from African biomass burning [9,10].

Recent global assessments provide the large-scale context against which regional records such as ours must be interpreted. Joshi et al. [11] analysed CO at 850 hPa and reported a widespread global decline, which they attribute to the adoption of cleaner combustion technologies together with a reduction in fire activity in the major burning hotspots; they also emphasise that CO is not a greenhouse gas itself but alters the chemistry of methane and ozone and so affects radiative forcing indirectly. Pathak et al. [12] examined the spatial heterogeneity of atmospheric CO during the COVID-19 lockdown and found column reductions of roughly 5–10% over regions of intense anthropogenic activity such as East China and the Indo-Gangetic Plain, but no comparable reduction where wildfires and agricultural waste burning dominate, and no significant change at stratospheric altitudes. Taken together, these studies establish that the global and near-surface tendency of CO is downward and that this tendency is strongly heterogeneous, being governed by the balance between technological emission controls and fire activity in each source region. Our results are consistent with that picture in the background air of the North Pacific upper troposphere, where the monthly minimum CO shows a weak decline, but they also show that the most polluted air masses over the mid-latitude North Pacific behave differently, which we discuss in the Results.

While in-situ observations of atmospheric gas-phase pollution levels in the upper troposphere over the North Pacific are scarce and challenging, satellite remote sensing techniques offer passive observations in near and thermal infrared wavelengths to infer total column density of gas-phase molecules [13]. However, obtaining good vertical resolution of CO in the upper troposphere remains a challenge. Ozone (*O*_3_) measurements by ozonesonde provide valuable vertical profile data but are limited to ozone [14].

The global atmosphere has been continuously impacted by significant inputs of ground-level anthropogenic pollutants since the industrial revolution [15,16]. Routine and long-term in-situ measurements of Asian air pollutants over the North Pacific upper troposphere are rare but crucial for understanding anthropogenic impacts in this region. In this work, we present routine in-situ measurements of CO from the PGGM (Pacific Greenhouse Gases Measurement)/IAGOS (In-service Aircraft for a Global Observing System) air-based measurement project conducted during the period 2012–2018 [2]. Combining these measurements with previous in-situ data collected in various years between 1991 and 2013, we observed a significant increase in anthropogenic pollutant CO in the upper troposphere over the North Pacific [17]. These elevated levels, predominantly observed during the summer months of July and August, indicate effective vertical transport of ground-level anthropogenic pollutants [18–24].

Two considerations motivate the specific focus of this study on the North Pacific upper troposphere. First, the region is the dominant export pathway by which Asian anthropogenic pollution enters the global background atmosphere: air lifted out of the East Asian boundary layer by frontal and warm-conveyor-belt systems is carried eastward across the basin, so the composition of the North Pacific upper troposphere integrates the emissions of the world’s most rapidly industrialising region. Second, the region is chronically under-observed. There are almost no long-term surface monitoring sites over the open ocean, satellite retrievals have limited vertical resolution in the upper troposphere, and dedicated research aircraft campaigns sample only a few weeks at a time. Long-term in-situ measurements at cruise altitude are consequently the only observational record capable of resolving multi-decadal change in this part of the atmosphere.

Against this background, the present study makes three contributions that are not available from any single existing dataset. (i) It assembles a multi-decadal synthesis spanning 1991–2018, joining the PGGM/IAGOS commercial-aircraft record to the earlier NASA PEM-West, TRACE-P, HIPPO and ATom campaigns and to the NOAA Mauna Loa flask record, so that campaign-era and routine-monitoring-era observations are placed on a common footing. (ii) It performs a cross-platform consistency assessment, quantifying how in-situ aircraft data, MOPITT satellite retrievals and a global chemistry transport model differ in the same air masses, and thereby establishing how far each platform can be trusted for trend detection in the upper troposphere. (iii) It attributes the resulting trends regionally, separating the mid-latitude and high-latitude North Pacific and contrasting both with the North Atlantic, and it distinguishes trends in the most polluted air masses from trends in background air by analysing the monthly maximum, minimum, percentile and mean statistics separately rather than the mean alone.

In the following, we present new insights into air pollution in the upper troposphere, highlighting the importance of routine in-situ measurements for understanding the impacts of anthropogenic emissions on atmospheric chemistry and dynamics. Emissions can alter the vertical distribution of key chemical species, thereby modifying radiative balances and influencing convection and atmospheric stability. These changes affect vertical transport processes and overall circulation patterns in the atmosphere. For instance, controlling CO emissions not only reduces pollutant levels but also helps mitigate their potential effects on atmospheric dynamics. By combining observational data with advanced modeling techniques, we can track and predict the spatial and temporal variations of air pollutants, thereby informing effective mitigation strategies (such as CO emission control measures) and policies like regulatory emission standards and air quality management programs). Table 1 shows a summary of studies on long-term atmospheric CO trends, including data sources, periods, and key findings.

**Table 1:** Summary of studies on long-term atmospheric CO trends, including data sources, periods, and key findings.

Study (Reference)	Data Used	Data Period	Key Findings
Duncan et al. [25]	NOAA/CMDL surface network, NASA GTE aircraft data (PEM-West A, PEM-West B, PEM-Tropics); MAPS satellite data	1988–1997	Emissions hardly change from 1988 to 1997, as increases in Asia are offset by decreases elsewhere.
Worden et al. [26]	MOPITT, AIRS, TES, IASI satellites; NOAA surface sites	2000–2010	A –1%/yr decline in total column CO, including a previously unreported decrease over Eastern China. Biomass burning explains some interannual variability, but anthropogenic factors require further study. The trends appear in both hemispheres, with a stronger signal in the Northern Hemisphere.
Yin et al. [27]	Satellite data (MOPITT)	2000–2010	Decadal trends in global CO emissions, highlighting the role of anthropogenic and biomass burning sources.
Osman et al. [28]	MOZAIC/IAGOS aircraft measurements, MOPITT satellite data	2001–2012	Upper tropospheric CO variability is influenced by surface emissions, transport, and chemical loss processes; differences of −20% in CO volume mixing ratio.
Zheng et al. [29]	Emission inventories, atmospheric observations in China	2010–2017	From 2010 to 2017, China’s CO emissions fell by 27%, largely driven by stricter pollution controls on power plants and industries. Emissions cuts accelerated after 2013, leading to a 23% CO reduction from 2013 to 2017, confirming the effectiveness of China’s Clean Air Action.
Joshi et al. [11]	TES reanalysis CO at 850 hPa; emission inventories; fire counts	2005–2018	Global CO declines, driven by the adoption of cleaner combustion technologies and by reduced fire activity in the burning hotspots. CO is not a greenhouse gas but changes the chemistry of *CH*_4_ and *O*_3_ and so indirectly affects radiative forcing.
Pathak et al. [12]	Satellite CO columns and profiles	2020 (lockdown vs. pre-lockdown)	CO columns fell by 5–10% over regions of high anthropogenic activity (East China, Indo-Gangetic Plain, North America, parts of Europe and Russia) during the COVID-19 lockdown, but not over regions dominated by wildfires and agricultural waste burning; no significant change at stratospheric altitudes. Emission reduction alone is therefore not sufficient to improve air quality where fires dominate.
Smoydzin and Hoor [30]	MOPITT, MPTRAC model, ERA-Interim reanalysis	2016–2018	China is the main year-round CO source, with air masses lifted into the free troposphere via a frontal system. NE Asia (via warm conveyor belts) contributes in winter, spring, and summer, while India dominates in spring and summer, and SE Asia in spring.

## Data and methods

In this work we utilize a diverse range of CO measurement datasets to analyze long-term trends in various atmospheric regions. The datasets include: PEM-West A (1991) and PEM-West B (1994): Grounded in targeted aircraft campaigns focusing on Pacific outflow pollution events, utilizing Non-Dispersive Infrared (NDIR) Analyzers. TRACE-P (March–May 2001): Conducted during spring to capture peak Asian pollution transport, employing Non-Dispersive Infrared (NDIR) Analyzers. HIPPO (2009, 2010, 2011): Multiple pole-to-pole campaigns providing high-precision CO measurements using Quantum Cascade Laser Spectrometers (QCLS). ATom (2016, 2017, 2018): Continuous airborne observations capturing a wide range of atmospheric conditions, utilizing Wavelength-Scanned Cavity Ring-Down Spectroscopy (WS-CRDS) (Picarro G2401). IAGOS (1997–ongoing): In-service aircraft data offering consistent, high-precision CO measurements globally with Non-Dispersive Infrared (NDIR) Analyzers. MOPITT (1999–ongoing): Satellite-based measurements using Infrared and Microwave Radiometers with layer-specific uncertainties. Mauna Loa Observatory (1989–ongoing; MLO; $19.536^{\circ }N$, $155.576^{\circ }W$, and at an altitude of about 3397 m above sea level): Ground-based flask samples were measured by gas chromatography. The flask sample data version 2003-10-02.1147 was used for this work. Each dataset’s measurement period, method, error values, and validation references are summarized in Table 2 shown below.

**Table 2:** CO Measurement Datasets with Combined Errors and References.

Dataset	Measurement Period	Measurement Method	Error Values	Combined Error (%)	References
Mauna Loa	1989 – present	GC (flask);	Accuracy: 2.3 ppb to 3.1 ppb (GC) 10 ppb (NDIR);	±2-5%	[43,47,48]
NASA PEM-WEST A	Sept–Oct 1991	TDL on NASA DC-8 aircraft	Precision: 1%; Accuracy: 1%	±1%	[39]
NASA PEM-WEST B	Feb–Mar 1994	TDL on NASA DC-8 aircraft	Precision: 1%; Accuracy: 1%	±1%	[40]
MOPITT	1999 – present	Satellite Infrared/Microwave Radiometers	Upper Troposphere: 10–20%; Lower Troposphere: 5–10%	Up to 20%	[26,62–65]
HIPPO	2009–2011	QCLS	Precision: ± 0.15 ppb; Mean Difference: ~2 ppb	±2.15%	[23,66]
IAGOS	1997 – present	IR gas-filter correlation	Precision: ± 1–2 ppb; Systematic: ± 2%	±5%	[67–69]
TRACE-P	Feb–Apr 2001	TDLAS on DC-8 and P-3B	Accuracy: 1%	1%	[21]
ATom	2016–2018	OCLS	± 0.15 ppb precision; accuracy 3.5 ppb	~ 3.65 ppb	[70,71]

Note: Combined Error (%) represents the sum of precision and accuracy uncertainties. “Up to” indicates the maximum combined error for datasets with varying error contributions.

These datasets do not play equal roles in the analysis, and we distinguish them explicitly here. The *primary* datasets are IAGOS and MOPITT. IAGOS provides the in-situ backbone of the study: routine, calibrated, high-frequency CO measurements at cruise altitude along fixed flight corridors, together with vertical profiles during ascent and descent, which constrain both the absolute CO abundance and its vertical structure in the upper troposphere over the North Pacific. MOPITT provides the spatially continuous context that aircraft cannot: global coverage of CO columns and coarse profiles, which we use to establish where the North Pacific trends sit within the global pattern and to test whether the aircraft sampling is regionally representative. The IMS chemistry transport model is the third primary component, used to separate the contributions of chemical production and loss from those of transport. The *supplementary* datasets serve validation and extension rather than trend detection in their own right. PEM-West A, PEM-West B and TRACE-P extend the record back to the pre-IAGOS era and anchor the early 1990s and 2001 baselines, but their coverage is a few weeks per campaign and they are used for comparison of distributions, not for fitting trends. HIPPO and ATom constrain free-tropospheric and remote-Pacific CO with high-precision instruments and are used to check the IAGOS values in overlapping air masses. The Mauna Loa flask record provides an independent, continuously calibrated ground-truth time series against which both the model and the satellite retrievals are evaluated. The temporal coverage used in this work ends as follows: IAGOS CO measurements from the PGGM flights, July 2012 to December 2018; MOPITT Version 8 Level 3 monthly gridded product, March 2000 to May 2021; NOAA Mauna Loa flask CO, 1989–2021; NASA campaign data as listed in Table 2; and the IMS simulations covering September 1948–August 1978 and September 1984–August 2003. The choice of 1991–2018 as the analysis window is deliberate rather than incidental. First, it defines a homogeneous multi-decadal epoch that brackets the major policy transition in the dominant upwind source region, spanning the period of rapid growth in East Asian emissions and the years immediately following China’s 2013 Clean Air Action Plan, so that the response of the upper troposphere to that transition can be examined within a single consistent record. Second, it matches the coverage of the EDGAR v6.1 gridded inventory (1970–2018) used throughout the model analysis, so that observed trends and emission trends are compared over identical periods. Third, it preserves cross-instrument calibration continuity: extending the record past 2018 would require changing MOPITT product version and would introduce the sharp, pandemic-driven discontinuity in commercial flight density that affects IAGOS sampling from 2020 onward. Post-2018 trends are therefore better addressed by a dedicated follow-up study using a sampling framework designed for the altered flight matrix.

### In situ observations

#### PGGM/IAGOS.

Fig 1(a) depicts the flight routes of three routine in-service commercial passenger Airbus aircraft operated by China Airlines (CAL) and equipped with the IAGOS Package 1 instruments for in-situ measurements from 2012 to 2018 [2,17,31]. These flight routes span various regions, covering heavy emission areas over East Asia [22], upwind and downwind of Asian emission areas over the Euro-Asia continent, the North Pacific, tropical northwestern Pacific regions, as well as flights to the southern hemisphere, including Australia and New Zealand [32].

**Fig 1:**
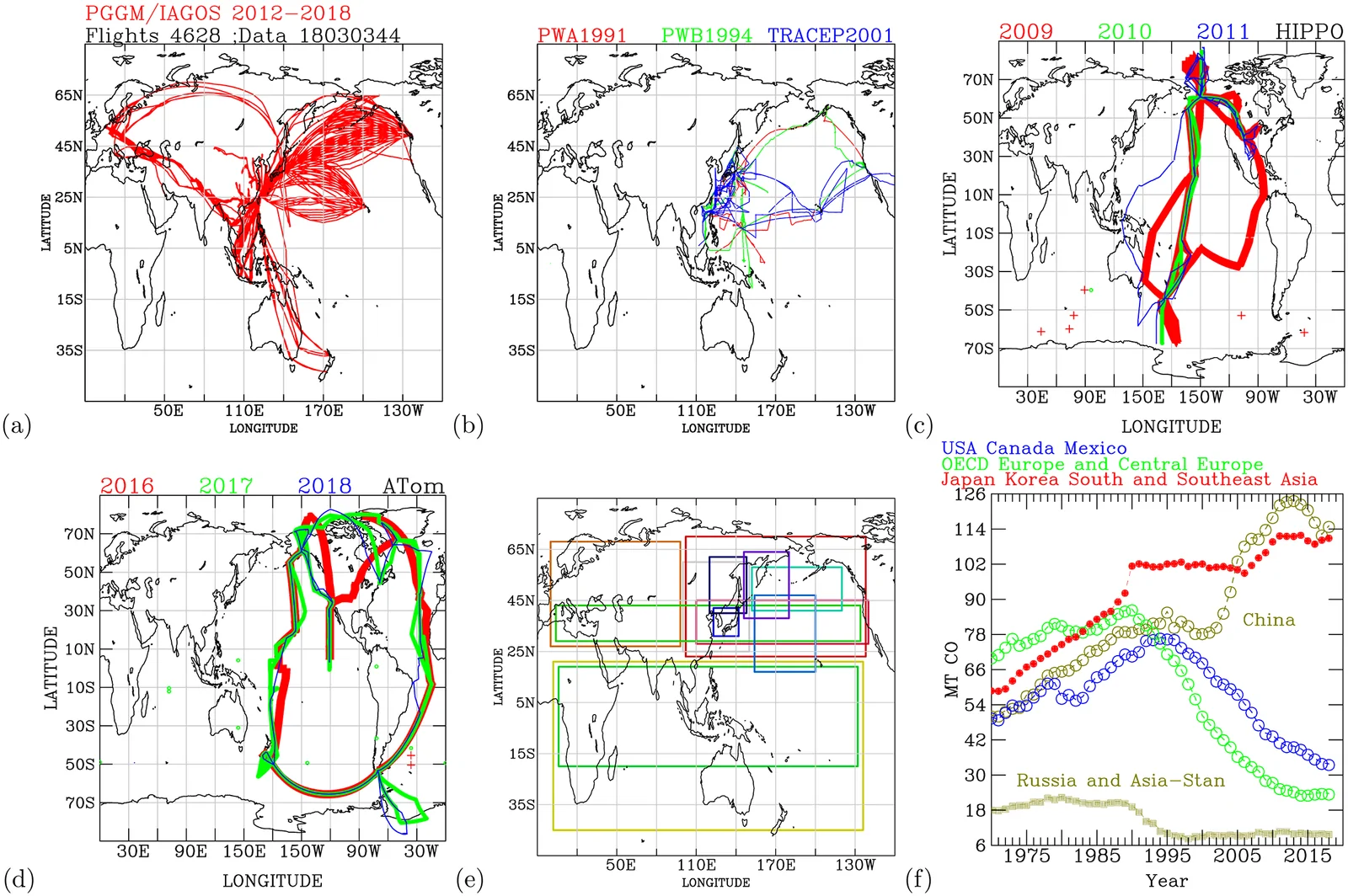
The flight routes of the measurement aircrafts and analysis domains. (a) The flight routes of the PGGM/IAGOS aircrafts during 2012-2018. (b) The flight routes of the NASA PEM-WEST A in 1991 (red), the PEM-WEST B in 1994 (green), and the TRACE-P in 2001 (blue). (c) The flight routes from the NASA HIPPO aircraft measurements during 2009 (red), 2010 (green), and 2011 (blue). (d) The flight routes from the NASA ATom aircraft measurements during 2016 (red), 2017 (green), and 2018 (blue). (e) The analysis domains. The area with intensive surface cyclogenesis (dark blue), and its downwind region to the central Pacific (purple), and over the entire North Pacific (sky blue). The northern hemisphere warm conveyor belt (light green in the NH), and its subdomains, including downwind of the Asian continent, and across the entire North Pacific(magenta); in the exit of airs from Asia content over the Japan and Korea region (blue); and further downwind, across central Pacific (navy blue). The western part of the Euro-Asia continent, including part of the warm conveyor and its northern latitudes (brown). The eastern part of the Euro-Asia continent and the North Pacific (red). The tropical regions and the southern hemisphere (dark green), and the tropical regions (fresh green). (f) Time-series technological emissions of carbon monoxides estimated by the EDGAR version 6.1 for USA, OECD and Central Europe, Asian countries excluding China, China, and Russia and Central Asian countries.

It is noteworthy that the IAGOS project is a continuation of the MOZAIC project, which commenced in 1994 [33,34]. The innovative aspect of the MOZAIC project lies in its utilization of five in-service commercial passenger Airbus A340-300 aircraft to routinely collect air pollutants in the upper troposphere during commercial passenger service flights. Unlike dedicated research aircraft, commercial passenger aircraft operate extensively, allowing for data collection during take-off, cruising, and landing stages, thus providing abundant data opportunities [17]. This stands in stark contrast to research aircraft, which typically have limited operational windows spanning only a few days.

#### NASA GTE, HIPPO, and ATom aircraft measurements.

Thanks to the visionary, courageous, and painstaking effort of David Keeling and collaborators, the Mauna Loa *CO*_2_ data provide long-term, in-situ, and consistent monitoring of atmospheric *CO*_2_ since March 1958 [35,36]. These data serve as a crucial reference for quantifying the current status of atmospheric *CO*_2_ in a climate characterized by rising temperatures and continuously increasing anthropogenic emissions [1].

CO is not itself a greenhouse gas in the radiative sense, but it is a short-lived climate-relevant pollutant whose indirect forcing is far from negligible. As the dominant sink of the hydroxyl radical, CO controls the oxidising capacity of the troposphere and thereby modulates the lifetime of methane and the photochemical production of tropospheric ozone, both of which are greenhouse gases; it is additionally oxidised to *CO*_2_ itself. Partitioning radiative forcing by emitted compound rather than by resulting concentration, the IPCC Fifth Assessment Report attributes to CO emissions a radiative forcing of 0.23 [0.16 to 0.30] W *m*^–2^ over 1750–2011, delivered through the resulting changes in *CO*_2_, *CH*_4_ and *O*_3_, with medium confidence [37]. This exceeds the forcing attributed to *N*_2_*O* (0.17 [0.13 to 0.21] W *m*^–2^) and to the halocarbons (0.18 [0.01 to 0.35] W *m*^–2^), and amounts to roughly one seventh of that attributed to *CO*_2_ emissions themselves (1.68 [1.33 to 2.03] W *m*^–2^). Long-term monitoring of CO is therefore climatically as well as chemically motivated [11]. For such short-lived climate-relevant species, long-term measurements downwind of heavy surface emission areas are rare but essential for understanding changes in air pollutants over the North Pacific upper troposphere. During the period 1970–1994, when technological emissions grew significantly year by year, the only in-situ measurements in the upper troposphere over the North Pacific were conducted as part of NASA’s Global Troposphere Experiments (GTE) [18,38].

Fig 1(b) illustrates the flight routes of NASA’s PEM-WEST A in 1991 [39], PEM-WEST B in 1994 [40], and TRACE-P in 2001 [21]. The PEM-WEST A and B flight routes focused on regions west of $140^{\circ }E$ over the western North Pacific, near the East Asia emission areas. These flights provided valuable data on air pollutants over the North Pacific upper troposphere immediately downwind of East Asia emission areas, especially during crucial periods such as 1991 and 1994, which marked the end of steady CO emission growth and with significant growth thereafter, particularly from China [22]. The visionary PEM-WEST measurements offer valuable data for comparison with later in-situ measurements from projects like IAGOS and ATom.

Fig 1(c) depicts the flight routes from NASA’s HIPPO aircraft measurements during 2009, 2010, and 2011 [23], while Fig 1(d) shows the flight routes from NASA’s ATom aircraft measurements during 2016, 2017, and 2018 [24]. The HIPPO measurements focused on regions close to the International Dateline (ID) and longitudes east of it in the North Pacific upper troposphere. Similarly, the ATom measurements over the North Pacific focused on regions similar to those covered by HIPPO flights.

Based on these flight routes, we categorized the data into regions for analysis, as shown in Fig 1(e). These regions are characterized by key dynamical processes responsible for transporting ground-level air pollutants to the upper troposphere. They include regions of intensive surface cyclogenesis and their downwind areas, the northern hemisphere warm conveyor belt and its subdomains, areas of air exit from the Asian continent, as well as regions across the Euro-Asia continent and the North Pacific.

The PEM-West A, PEM-West B, and TRACE-P missions provided targeted observations of the Pacific atmosphere during distinct seasons and years. While limited in temporal and spatial coverage, these missions were designed to capture representative atmospheric conditions, particularly in regions of enhanced transport from continental sources [21,41]. To ensure the representativeness of these datasets, we compared their statistical distributions with more continuous observations from IAGOS and MOPITT. This analysis confirmed that the PEM and TRACE-P data align with long-term trends in CO over the Pacific, supporting their inclusion in this study.

The sparse nature of the PEM-West and TRACE-P datasets poses limitations in terms of temporal coverage; however, their strategic design ensures that the data are representative of atmospheric conditions during the periods of interest. Validation efforts comparing these datasets with more continuous observations (e.g., IAGOS and MOPITT) confirm their consistency with broader trends. The unique vertical profiles provided by these missions complement satellite and surface-based observations, offering critical insights into transport processes and chemical evolution in the remote Pacific atmosphere.

The PEM-West and TRACE-P campaigns provide a unique dataset for studying elevated pollution cases, which are less frequently observed in long-term monitoring datasets such as IAGOS and MOPITT. When compared with these more continuous datasets, the PEM and TRACE-P measurements align well during periods of elevated pollution, highlighting their representativeness for analyzing extreme pollution events. This consistency confirms the value of these targeted missions for advancing our understanding of pollution transport and chemistry.

By focusing on periods of enhanced pollution, the PEM and TRACE-P datasets complement continuous observations from programs like IAGOS, MOPITT, and Mauna Loa, which capture a mix of polluted and clean conditions. This distinction allows the PEM and TRACE-P datasets to serve as benchmarks for studying extreme pollution events, including their transport, chemical evolution, and impact on regional and global atmospheric composition. Their inclusion in this study provides critical insights that would not be evident from mean values alone.

#### Mauna Loa surface long-term measurements.

The Mauna Loa monitoring site, operated by the Global Monitoring Laboratory (GML), National Oceanic and Atmospheric Administration (NOAA), provides high-quality, long-term, in-situ flask measurements of atmospheric CO [42–48]. These measurements play a crucial role as a standard reference for evaluating long-term CO trends observed from satellite measurements by instruments like MOPITT and simulations by models such as the Integrated Modelling System (IMS) model.

### MOPITT satellite remote sensing measurements

Satellites serve as valuable tools for remote monitoring of CO on a continuous and global scale from low Earth orbit. The Measurements of Pollution In The Troposphere (MOPITT) instrument onboard NASA’s Earth Observing System Terra has been monitoring CO globally since March 2000 [49,50]. The openly available MOPITT data are extensively utilized in documenting CO variations associated with various atmospheric processes. In this study, we leverage MOPITT data as a consistent reference to assess the long-term trends of CO in the troposphere.

Worden et al. [26] focused on satellite-based CO trends, particularly from the MOPITT instrument, and its data characteristics. Worden et al. identified a modest decreasing trend of approximately −1% per year in the Northern Hemisphere total column CO, based on satellite observations from 2000 to 2011. This trend is less significant in the Southern Hemisphere, suggesting regional differences in emission sources and atmospheric dynamics. Regionally, decreases are observed in the USA, Europe, and Eastern China, with the latter being a notable finding, as it was previously unreported. This is particularly interesting given Eastern China’s industrial activity, suggesting potential shifts in emission controls or economic activities. The unexpected decline in Eastern China warrants further investigation.

The MOPITT data used in this study were obtained in 2023 from NASA Langley Research Center official repository (https://l5ftl01.larc.nasa.gov/ops/misrl2l3/). Specifically, we used the MOP03JM product (version 008) Level 3 dataset (e.g., file MOP03JM-200003-L3V95.6.3.he5 for March 2000) [51]. Currently, the website to download MOPITT data is https://asdc.larc.nasa.gov/data/MOPITT (as of 19 June 2025). Following is an example link to download the data https://asdc.larc.nasa.gov/data/MOPITT/MOP03JM.008/2000.03.03/MOP03JM-200003-L3V95.6.3.he5 (as of 19 June 2025). Notice that the MOPITT data is openly available, but registration at NASA Earthdata (https://urs.earthdata.nasa.gov/home as of 19 June 2025) is required to download the MOPITT data.

This Level 3 product is provided on a global $1^{\circ }$x$1^{\circ }$ latitude–longitude grid, which ensures a consistent horizontal resolution suitable for regional and global analyses. In addition, the retrieval algorithm employs an optimal estimation approach that yields effective vertical resolution corresponding to two independent layers–-typically representing near-surface and free-tropospheric CO concentrations–-as determined by the associated averaging kernels. There are nine vertical levels in the data (900hPa, 800hPa, 700hPa, 600hPa, 500hPa, 400hPa, 300hPa, 200hPa, and 100hPa). The altitudes at each pressure level were estimated by the barometric equation, which give altitudes of 967m, 1829m, 3048m, 4572m, 5050m, 6100m, 9144m, 12192m, and 15240m. This pressure-height relationship give a scale height of 6500m. This means that on average, every increase in height by about 6500 m causes the pressure to drop by a factor of e (approximately 2.718). It is important to note that when we compute the “local” scale height between successive data points, we get somewhat different values. This variation is expected because in the real atmosphere the temperature (and hence the scale height) is not constant with height. For example, between 900 hPa and 800 hPa the effective scale height is different than between 200 hPa and 100 hPa.

In tropical regions the atmosphere is generally warmer. This higher temperature increases the scale height (often 7000–8000 m), so for a given pressure level (say 500 hPa) the atmosphere is “thicker” and the level is found at a higher altitude than what a constant 6500 m scale height would predict. Mid-latitude regions (where most of the IAGOS measurements were taken) typically have moderate temperatures. In these areas, the effective scale height is closer to the average value (around 6500 m), so the estimated heights using the simple exponential formula tend to agree reasonably well with observed values. At high latitudes, the atmosphere is colder. A lower temperature reduces the scale height (often around 6000 m or even less), which means that pressure surfaces occur at lower altitudes than the constant 6500 m model would indicate.

We estimate that by using a midlatitude (6.5 km) scale height uniformly, we typically underestimate the altitude of a given pressure surface by about 1 km over the tropics. In high latitudes, we tend to overestimate it by a similar amount (about 1 km). These differences are approximate and depend on the specific pressure level considered–-the logarithmic relationship means that discrepancies become larger at higher altitudes (or for larger pressure ratios).

We note that the MOPITT V9 data [52] was peer-reviewed and published relatively recently, on 19 April 2022. Validation of V9 using in situ CO profiles from aircraft in various contexts shows retrieval biases typically within ±5%, comparable to those for the V8 product [52]. This observation aligns well with the timeline of our study and strengthens the justification for using V8 data in our manuscript. We use MOPITT’s Thermal-Infrared (TIR)-only retrievals, which are sensitive to upper tropospheric CO–-our target region. V9’s improvements, such as refined Near-Infrared (NIR) calibration, primarily affect total column CO and are less relevant to TIR-only profiles. V9 improves cloud detection, notably increasing daytime retrieval coverage over land [52]. we acknowledge V9’s availability and recommend its consideration for future studies, particularly those targeting land-based regions or total column CO.

### EDGAR technological emission estimates

An openly available and widely used source for estimating gridded anthropogenic emissions in global chemistry and climate models is the Emissions Database for Global Atmospheric Research (EDGAR) from the European Commission [53].

In this study, we utilize EDGAR estimates to track variations in technological emissions of CO during the period 1970–2020. Fig 1(f) illustrates time-series technological emissions of carbon monoxide estimated by EDGAR version 6.1 for various regions including the United States (USA), OECD countries, Central Europe, Asian countries excluding China, China, and Russia and Central Asian countries. EDGAR data play a crucial role in revealing the variations of technological emissions in our analysis.

It is worth noting that accurate estimates of anthropogenic emissions are essential for quantifying the discrepancies between observed rising temperatures and anthropogenic emissions, as highlighted in the United Nations Environment Programme Emissions Gap Report 2023 [1].

### IMS global tropospheric chemistry model

The three-dimensional chemistry transport model IMS is employed in this study to simulate long-term CO trends and budgets in the troposphere. The IMS model encompasses full tropospheric chemistry, with particular focus on the conversion of hydrocarbons to CO [54], along with a comprehensive suite of anthropogenic and natural emissions [55]. Validation of IMS model results has been conducted against observational data [56–58]. Additionally, the IMS model has been utilized to investigate various phenomena, including the impact of stratospheric ozone on tropospheric ozone [59] and the long-range transport of Asian dust over the North Pacific [60].

The IMS model uses 48x40 grid resolution, 19 vertical layers (surface to 10 hPa), driven by NCEP reanalysis [61], with a complete tropospheric chemistry. The model was configured for historical runs (1950s–2003) with largely constant anthropogenic emissions after 1990. We note that this approach cannot capture post-2003 emission changes and is thus used to explore pre-2003 variability and general dynamical processes, and to compare with Mauna Loa measurements.

In this study, we conducted two long-term simulations using the IMS for tropospheric chemistry. The IMS 1948–1978 simulation spans 30 years, commencing from September 1948 to August 1978, while the IMS 1984–2003 simulation covers 20 years, starting from September 1984 to August 2003. These simulations were performed with constant emissions from anthropogenic sources, while biogenic emissions were calculated online based on interactions among the atmosphere, ocean, and terrestrial biosphere [55,57].

The results from the IMS simulations were compared with in-situ measurements from IAGOS, satellite remote sensing measurements from MOPITT, and long-term in-situ measurements from Mauna Loa.

### Carbon monoxide datasets and uncertainties

This study utilizes a comprehensive compilation of atmospheric carbon monoxide (CO) measurement datasets to analyze temporal and spatial variations in CO concentrations. The datasets encompassed in this analysis are summarized in Table 2, which outlines the measurement periods, methodologies employed, associated error estimates, and relevant references for each dataset. We combines in situ, satellite, and ground-based CO measurements to assess multi-decadal trends in atmospheric composition. The integration of these datasets leverages their complementary strengths in spatial coverage, vertical resolution, and temporal continuity, while rigorously accounting for measurement uncertainties. Table 2 categorizes nine distinct datasets, each contributing uniquely to the overall understanding of atmospheric CO trends. Fig 2 is a timeline Infographic that clearly presents the measurement periods, methods, and error values for each CO dataset.

**Fig 2:**
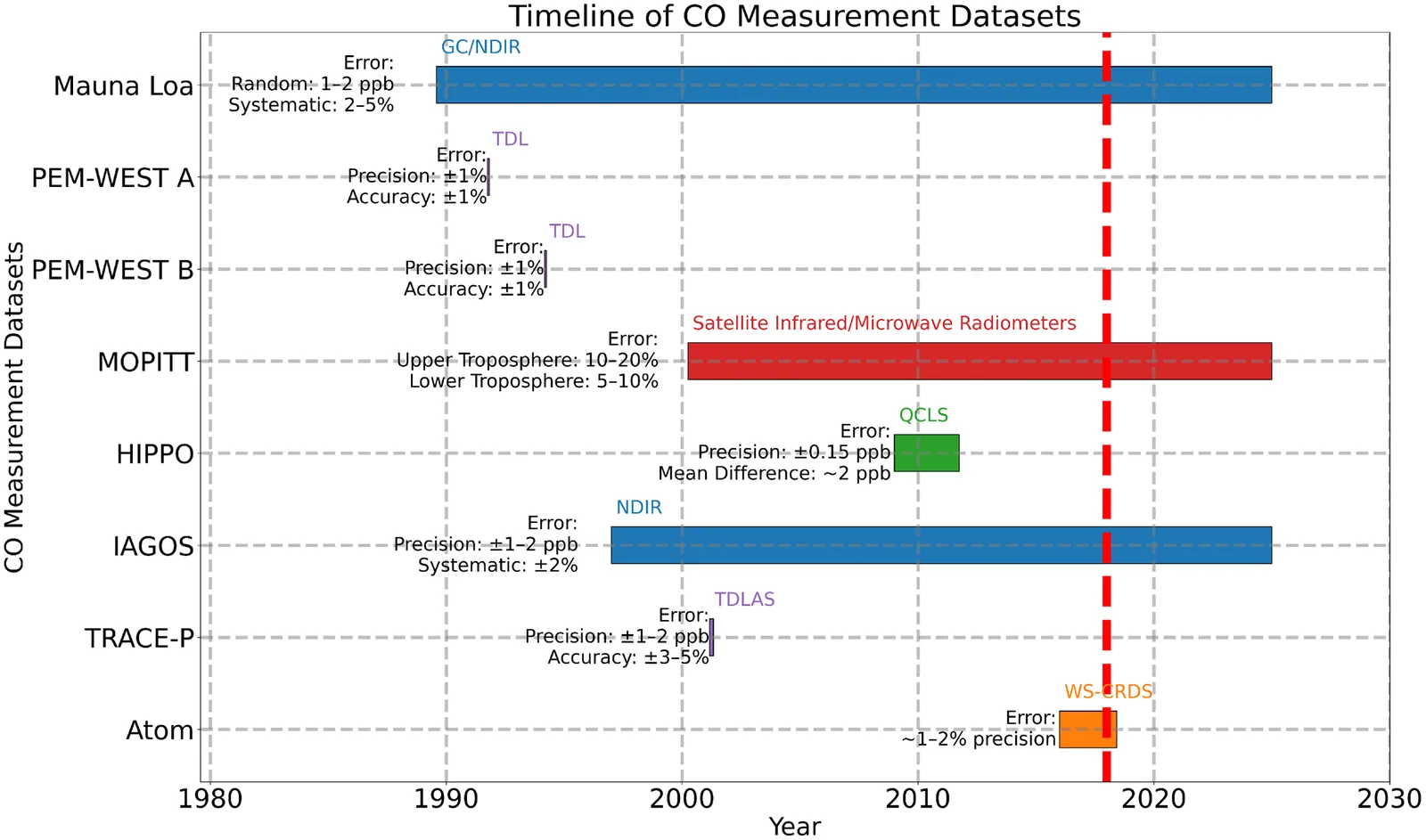
Timeline of CO Measurement Datasets. This infographic illustrates the chronological progression of various CO measurement datasets utilized in this study. Each dataset is represented by a horizontal bar (for multi-year measurements) or a point (for single-year measurements), color-coded by measurement method. Error values are displayed to the left of each dataset marker, providing insight into the associated uncertainties. A vertical red dashed line in 2018 indicate the end of data period used in this work.

Mauna Loa (1989–present): The Mauna Loa Observatory provides one of the longest continuous records of atmospheric CO. Flask measurements at Mauna Loa (1989–present) provide a ground-truth baseline for CO trends [42–46]. These data validate satellite and aircraft retrievals in the free troposphere.

NASA PEM-WEST A (1991) and B (1994): These campaigns involve CO measurements aboard NASA’s DC-8 aircraft utilizing NDIR analyzers and upgraded systems, respectively. PEM-WEST A, conducted from September to October 1991, reported precision errors of ±2 ppb and accuracy errors of ±5%, with additional sampling variability in plumes ranging from ±5–10%. PEM-WEST B, spanning February to March 1994, enhanced measurement precision to ±1.5 ppb and improved accuracy to ±4–5%. TRACE-P (2001): The Transport and Chemical Evolution over the Pacific (TRACE-P) mission integrates NDIR on the DC-8 aircraft and UV resonance fluorescence on the P-3B aircraft for CO measurements. Precision and accuracy errors are recorded at ±1–2 ppb and ±3–5%, respectively [21]. HIPPO (2009–2011): The HIAPER Pole-to-Pole Observations (HIPPO) program employs Quantum Cascade Laser Spectroscopy (QCLS) for high-precision CO measurements. The dataset boasts a precision of ±0.15 ppb and a mean difference of approximately 2 ppb [23,66]. The accuracy of CO measurements is further assessed by comparing QCLS data to independent measurements, such as NOAA’s Carbon Cycle Greenhouse Gases (CCGG) flask data collected during HIPPO. The mean difference between QCLS CO measurements and NOAA CCGG flask data over 59 HIPPO research flights was 2 ppb, indicating high accuracy relative to established standards [66]. ATom (2016–2018): The Atom project employs Whispering-Gallery Surface-Crystal Ring-Down Spectroscopy (WS-CRDS) for CO detection. The dataset demonstrates a precision of approximately 1–2% and an overall error of around 2%. These aircraft missions captured Asian pollution outflow over the Pacific. Their data anchor early 1990s–2000s baseline CO levels [21]. These short-term campaigns provide critical context for interpolating long-term trends.

MOPITT (2000–present) is a gas-correlation spectrometer on the Terra satellite using TIR (4.7 μm) and NIR (2.3 μm) channels to retrieve CO profiles via optimal estimation [72,73]. The joint TIR–NIR product differentiates lower (surface–500 hPa) and upper (500–200 hPa) tropospheric CO with precision of ~5–8% in the lower troposphere and ~10% in the upper troposphere, and bias errors generally under 15%. These global data fill gaps over oceans and remote continents where aircraft measurements (e.g., IAGOS) are sparse. Trend analyses using MOPITT (2000–2011) show column-CO precision ≤10% and biases <5% [26].

IAGOS (1997–present): The In-service Aircraft for a Global Observing System (IAGOS) project utilizes NDIR instruments to monitor CO across various flight routes. Precision errors range between ±1–2 ppb, and systematic errors are maintained at ±2%, as detailed by Gaudel et al. [68]. The IAGOS provides high-frequency CO measurements in the upper troposphere and lower stratosphere (UT/LS) from commercial aircraft since 1994. IAGOS measurements were validated against NOAA calibration scales [2]. Its routine profiling during takeoff and landing offers unparalleled UT/LS coverage over major flight corridors, serving as the backbone for trend analysis in this region. The IAGOS data used in this study were obtained from the IAGOS data portal [69].

Precision errors refer to the random uncertainties inherent in the measurement processes, typically expressed in ppb or percentage terms. High precision indicates consistency across repeated measurements. Accuracy errors denote systematic uncertainties that can cause measurements to deviate consistently from the true value. Accuracy is crucial for ensuring that the data reflects actual atmospheric conditions. Accurate error characterization is pivotal for the reliability of atmospheric CO measurements. The combined error percentages in Table 2 representing the aggregation of precision and accuracy uncertainties for each dataset. This aggregation was performed using the root-sum-square (RSS) method [74], assuming independent error sources. By considering both random and systematic uncertainties, the combined error provides a comprehensive measure of each dataset’s reliability and precision.

Each dataset’s error metrics have been meticulously compiled from the respective studies, ensuring that the analysis accounts for both random and systematic uncertainties. For instance, the Mauna Loa dataset’s systematic error of ±2–3% (Andrews et al., 2014; 2017) is considered alongside its random precision errors to provide a comprehensive error profile.

The integration of these diverse datasets allows for a robust analysis of CO concentrations across different temporal and spatial scales. The methodologies employed by each dataset complement each other, offering a multifaceted view of atmospheric CO dynamics:

Temporal Coverage: The datasets collectively cover over three decades, from 1989 to present, enabling the examination of long-term trends and seasonal variations in CO levels.

Spatial Distribution: Including both ground-based observatories (e.g., Mauna Loa) and airborne platforms (e.g., NASA PEM-WEST, HIPPO, TRACE-P) ensures a comprehensive spatial representation, capturing both local and regional CO variations.

Measurement Techniques: The utilization of various measurement techniques (NDIR, QCLS, WS-CRDS) enhances the methodological diversity, allowing cross-validation of results and mitigation of technique-specific biases.

In the subsequent analysis, data from each dataset within the targeted geographic regions were aggregated into monthly bins for both trend estimation and inter-dataset comparison. For each calendar month we then computed the following statistics: the mean, the median (50th percentile), the 25th and 75th percentiles, the monthly maximum, and the monthly minimum.

### Analysis strategy and statistical methods

The overall objective of this work – detection and attribution of long-term CO change in the North Pacific upper troposphere – is addressed through four distinct subtasks, each with its own method.

(i) *Linear and perturbed trend detection.* For each region and each monthly statistic we fit a linear regression of CO against time. Because the airborne records are sparse and carry non-negligible measurement uncertainty, the regression is not applied to the nominal data alone. Instead we apply a 10,000-sample Monte Carlo perturbation framework, described in detail in the section on measurement uncertainties below: each monthly value is perturbed within its ± 7% measurement uncertainty, and 10,000 random samples are drawn from the resulting ensemble of possible regressions. This yields distributions of slope, intercept and P-value rather than single values, so that the reported trend significance reflects measurement error as well as scatter. Trends are reported in ppb *month*^–1^ and, where useful, converted to ppb *decade*^–1^, together with the 95% confidence interval and the P-value. A trend is described as statistically significant only where the 95% confidence interval excludes zero; where it does not, we report the sign of the point estimate and the position of the interval and describe the result as suggestive rather than significant.
(ii) *Seasonal cycle evolution.* Monthly composites are constructed separately for the upper and lower troposphere, and the seasonal structure is examined by season (DJF, MAM, JJA, SON) and by correlation between upper- and lower-tropospheric CO within each season, which isolates the periods in which vertical transport couples the two layers.
(iii) *Interannual variability.* Departures from the fitted trend are examined against known episodic drivers, in particular biomass burning years and the campaign periods sampled by PEM-West, TRACE-P, HIPPO and ATom.
(iv) *Spatial heterogeneity.* The analysis is repeated for the separate latitude bands $25^{\circ }N-45^{\circ }N$ and $45^{\circ }N-60^{\circ }N$ over the North Pacific and, for contrast, for the North Atlantic, so that trends are not aggregated across regions with different upwind sources and transport regimes.

A deliberate feature of the strategy is that trends are computed not only for the mean but separately for the monthly maximum, the 75th, 50th and 25th percentiles, the minimum and the mean. The most polluted air masses and the background air respond to different drivers, and analysing only the mean would conflate them.

## Results

We report the following metrics consistently throughout this section: the trend slope, given in ppb *month*^–1^ and, for comparison with the wider literature, in ppb *decade*^–1^; the 95% confidence interval $\left[CI_{lower},CI_{upper}\right]$ on that slope; the P-value from the 10,000-sample perturbation framework; the Pearson correlation coefficient R for the relationship being examined; and, where model or satellite data are compared with in-situ measurements, the mean bias and the correlation between the two. Each of the following subsections is organised in the same order: the analytical goal, the data and method used, the key result with reference to the relevant figure or table, and a brief interpretation.

In all figures showing in the following, where a correlation coefficient R is shown, it represents the Pearson correlation between the variable plotted on the y-axis and the variable plotted on the x-axis. For example, when the y-axis shows CO in the upper troposphere and the x-axis shows time, R reflects the correlation between CO concentrations and time, representing long-term trends. When the y-axis shows CO in the upper troposphere and the x-axis shows CO in the lower troposphere, R quantifies the relationship between CO concentrations at different altitudes. The specific variables and corresponding time periods used in the correlation calculations are indicated in each Figure caption.

### Long-term time-series CO and trends

Fig 3 illustrates long-term time-series CO measurements downwind of the Asian emission areas and over the North Pacific upper troposphere. The upper panels depict data from high latitudinal regions north of $45^{\circ }N$, spanning longitudes $150^{\circ }E$ to $140^{\circ }W$, and latitudes $45^{\circ }N$ to $60^{\circ }N$. The lower panels display data from lower latitudinal regions south of $45^{\circ }N$, covering longitudes $150^{\circ }E$ to $140^{\circ }W$, and latitudes $25^{\circ }N$ to $45^{\circ }N$ (the Northern Hemisphere warm conveyor belt, Fig 3(e)). The time-series data are stratified according to the statistics of all data collected in a given month: the maximum (leftmost), 75th percentile (second from left), 50th percentile (center), 25th percentile (second from the right), and the minimum (rightmost) observations.

**Fig 3:**
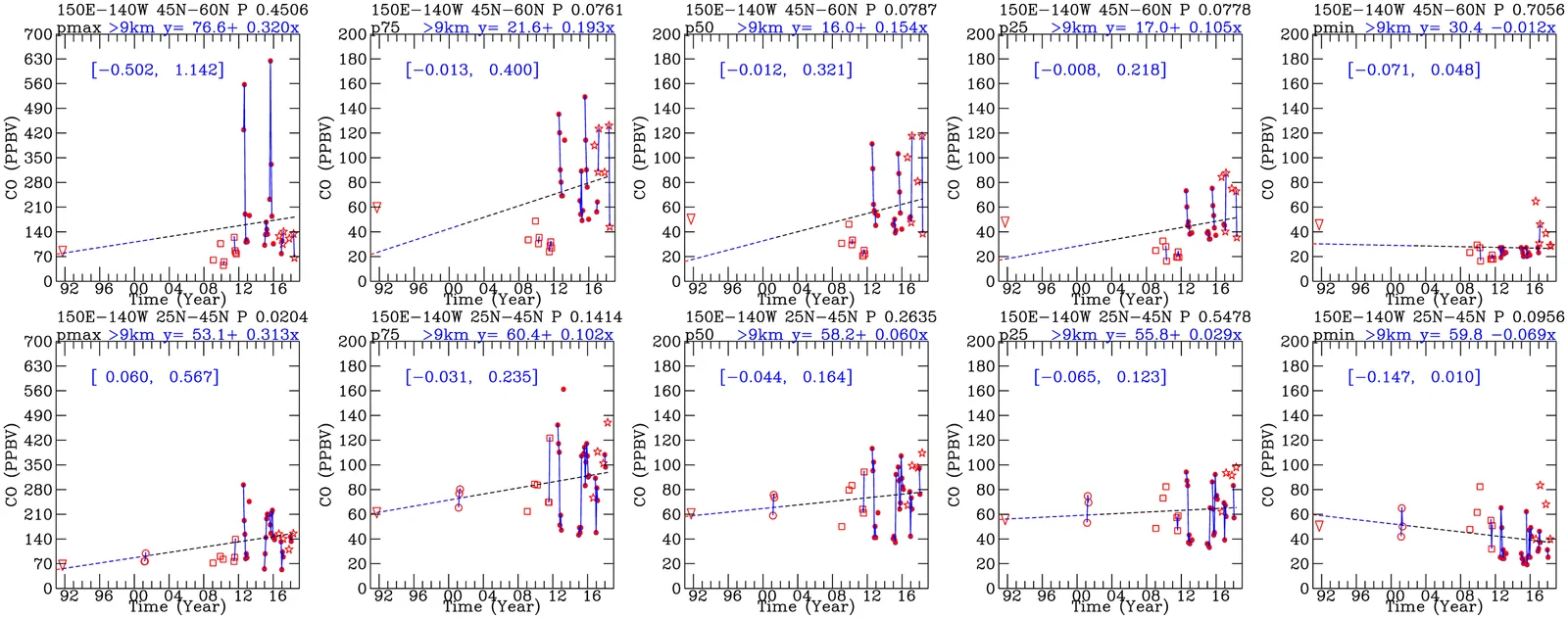
Time-series of long-term CO measurements downwind of the Asia emission areas and over the North Pacific upper troposphere during 1991-2018. The upper panels show data from high latitude regions north of $45^{\circ }N$, within longitudes $150^{\circ }E-140^{\circ }W$ and latitudes $45^{\circ }N-60^{\circ }N$. The lower panels show data from low latitude regions south of $45^{\circ }N$, within longitudes $150^{\circ }E-140^{\circ }W$ and latitudes $25^{\circ }N-45^{\circ }N$. The time-series data are stratified based on statistical distributions of all data collected in a given month, displaying: the maximum (leftmost), 75th percentile (second from the left), median (50th percentile, center), 25th percentile (second from the right), and minimum (rightmost) observations. The CO trends for each stratification are represented by a linear regression line (dashed blue). The regression equation is displayed at the top of each panel, while the statistical significance (P-value) is indicated in the top right of the figure title. The 95% confidence intervals of the CO trends are shown on each subplot. Specific datasets are marked as follows: Downward red triangles (1991): PEM-WEST A measurements; red circles (2001): TRACE-P measurements; red squares (2009–2011): HIPPO measurements; red stars (2016–2018): ATom measurements; small red circles (2012–2018): IAGOS measurements.

The maximum observed CO concentrations are higher over latitudes north of $45^{\circ }N$ (exceeding 280 ppbv) than over latitudes south of $45^{\circ }N$ (approximately 280 ppbv), indicating exceptionally high CO emission sources from biomass burning over higher latitudes such as Siberia [75].

The linear regression of the 75th percentile observations exceeds 90 ppbv in lower latitudes and falls below 90 ppbv in higher latitudes. For the 50th percentile observations, the linear regression is higher in lower latitudes (close to 80 ppbv) than in higher latitudes (around 70 ppbv). Regarding the 25th percentile observations, the linear regression for CO is higher (around 70 ppbv) in lower latitudes than in higher latitudes (approximately 50 ppbv).

Regarding the minimum observed CO levels, both higher and lower latitudes exhibit a decline. The lower latitudes contain higher CO levels (around 40 ppbv) compared to the higher latitudes (approximately 30 ppbv).

In summary, key findings from the in-situ aircraft measurements in the upper troposphere over the middle North Pacific, downwind of Asian emissions (see Fig 3), are as follows. Over the 27-year period from 1991 to 2018, all observed CO concentrations increased–-with the exception of the minimum and maximum levels. The minimum CO values, which representing background CO, decreased over time. In contrast, the 25th, 50th, and 75th percentiles of CO levels, which are influenced by industrial emissions, are higher at latitudes south of $45^{\circ }N$ compared to those north of $45^{\circ }N$. These findings are consistent with previous studies [76,77] that have documented the long-range transport of Asian pollutants across the North Pacific.

The 95% confidence intervals for the trends in CO concentrations indicate predominantly positive trends for the upper percentiles. Specifically, the confidence intervals are as follows: maximum, −0.502 to 1.142; 75th percentile, −0.031 to 0.235; 50th percentile, −0.044 to 0.321; and 25th percentile, −0.065 to 0.218. In contrast, the minimum CO levels exhibit a 95% confidence interval of −0.147 to 0.048, reflecting a predominance of negative trends. Overall, these results indicate that CO concentrations over the North Pacific have generally increased over the study period, except for the declining background levels.

Fig 3 combines the in-situ measurements from the airplanes, which are more accurate than the remote sensing measurements (Table 2). The maximum combined errors for the airplane measurements are less than ±7% (Table 2), here we test the impacts of these measurement errors on the linear regression of CO trends shown in Fig 3. For each monthly measurements X, we produce perturbed measurements of ±7%. Hence, there are three data points representing the range of possible measurements with respect to measurement errors: X + 7%X, X, and X-7%X. For two monthly measurements of *X*_1_ and *X*_2_, there are nine possible combinations of measurements with respect to ±7% of measurement errors. For n months of measurements, there are $3^{n}$ combinations of measurements for linear regression analysis. We typically have n = 30 months of data from airplane measurements. Hence the possible combination of linear regressions are $3^{n}$=3^30^. We take 10,000 random samples from $3^{n}$ of possible linear regressions with respect to measurement errors to study the impacts of the measurement errors on the linear regression trends and statistical significant levels shown on Fig 3.

Fig 4 presents a comprehensive analysis of long-term CO trends downwind of Asian emission areas and over the North Pacific upper troposphere, incorporating 10,000 linear regression calculations to assess trend variability and uncertainty. To address the potential errors in trend estimation, we stratified the CO time-series data based on key statistical distributions (maximum, 75th percentile, median, 25th percentile, minimum, and mean) to evaluate how different subsets of the dataset influence the computed trends. The figure systematically presents: (i) time-series trends based on multiple statistical approaches, (ii) the distribution of slopes, intercepts, and P-values from the regression analysis, and (iii) 95% confidence intervals (CI) to quantify the uncertainty in each trend estimate. The inclusion of these statistical measures ensures that our trend analysis is not solely dependent on a single regression outcome, but rather represents a robust assessment of possible long-term CO trends in the upper troposphere.

**Fig 4:**
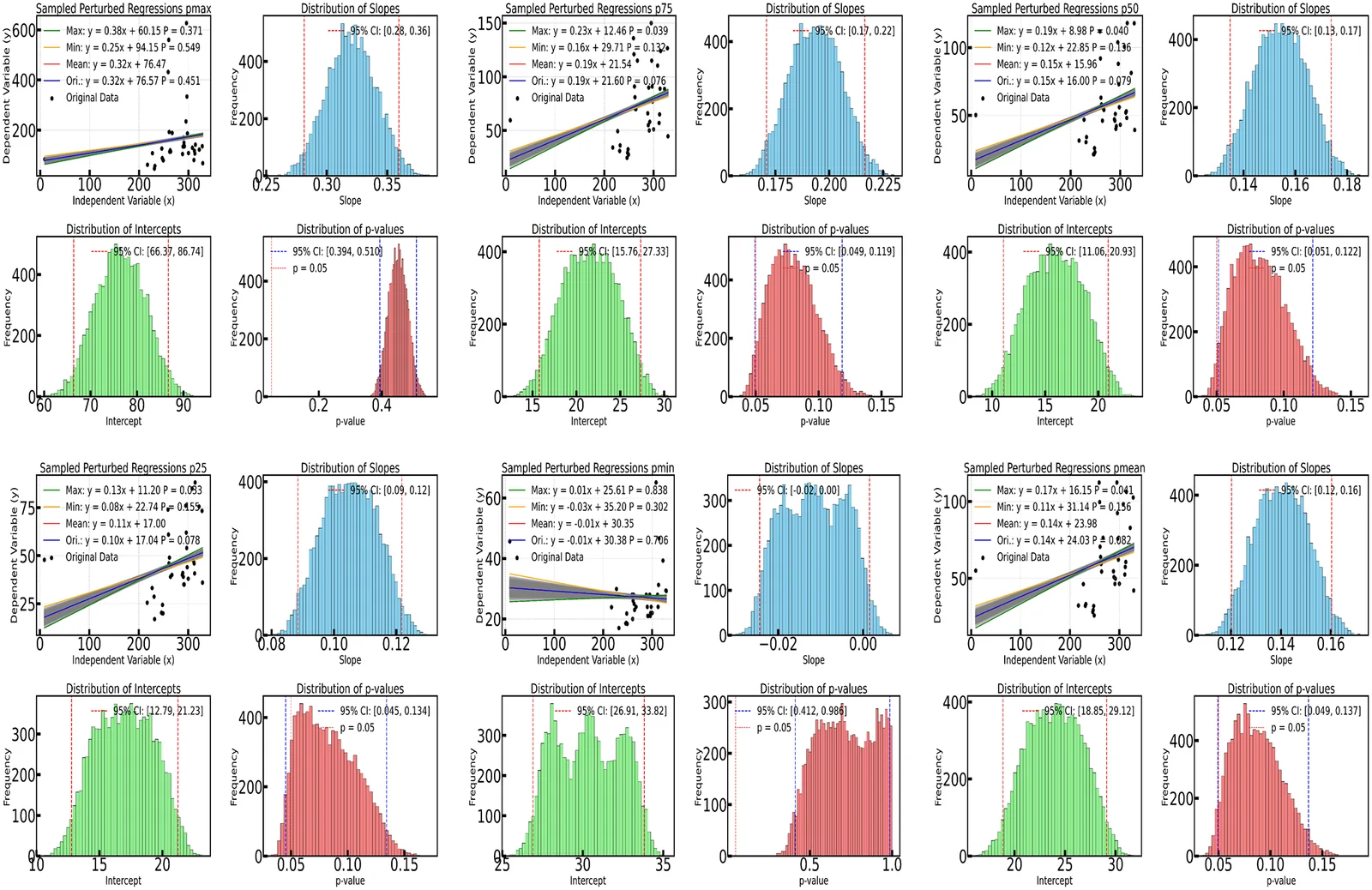
Time-series of long-term CO measurements and possible combinations of CO trends from linear regression analysis downwind of Asian emission areas and over the North Pacific upper troposphere, within longitudes $150^{\circ }E-140^{\circ }W$ and latitudes $45^{\circ }N-60^{\circ }N$. The time-series data and linear regression trends are stratified based on the statistical distributions of all data collected in a given month. Six statistical categories are presented in separate blocks of four panels: maximum values (top leftmost block), 75th percentile (top middle block), median (50th percentile) (top rightmost block), 25th percentile (bottom leftmost block), minimum values (bottom middle block), and mean values (bottom rightmost block). Each block of four panels provides a detailed breakdown of the CO trend analysis: Top Left Panel: Time-series data with all possible combinations of 10,000 linear regressions, including maximum, minimum, mean, and original regression equations, P-values from the original data; the original CO time-series data. Top Right Panel: Distribution of slopes from the 10,000 linear regression calculations. Bottom Left Panel: Distribution of intercepts from the 10,000 regression equations. Bottom Right Panel: Distribution of P-values from the 10,000 regression equations. Each distribution plot also displays the 95% confidence intervals (CI), providing a measure of statistical uncertainty.

Our perturbed test of 10,000 samples confirms that the statistical significance calculated from the original dataset is robust even when errors associated with measurements are taken into account. Specifically, if the original dataset shows a P-value below 5%, the errors included in the 10,000 perturbed samples also converge to a P-value below 5%, reinforcing the statistical significance of the observed trend. Conversely, if the original dataset yields a large P-value that is not statistically significant, the perturbed test with errors included also produces large P-values, demonstrating consistency across all samples. These additional analyses validate that when the original CO trend is statistically significant, the inclusion of measurement errors does not alter its significance, ensuring the reliability of our findings. This methodology directly addresses the need for error quantification, demonstrating the significance of our results while transparently acknowledging the inherent variability in atmospheric CO trends over time.

Fig 4 shows that CO trends north of $45^{\circ }N$ are statistically significant, with the highest frequency of P-values below 0.10, for the 75th percentile, 50th percentile, 25th percentile, and mean values. For CO trends south of $45^{\circ }N$ (Fig 5), the maximum and minimum values are statistically significant, with the highest frequency of P-values below 0.10. These stratified CO trends are consistent with the P-values shown in Fig 3. Hence, the trends of elevated CO exhibit a statistically significant increase, whereas the trends of background low CO show a statistically significant decrease over the North Pacific upper troposphere during the period 1991–2018.

**Fig 5:**
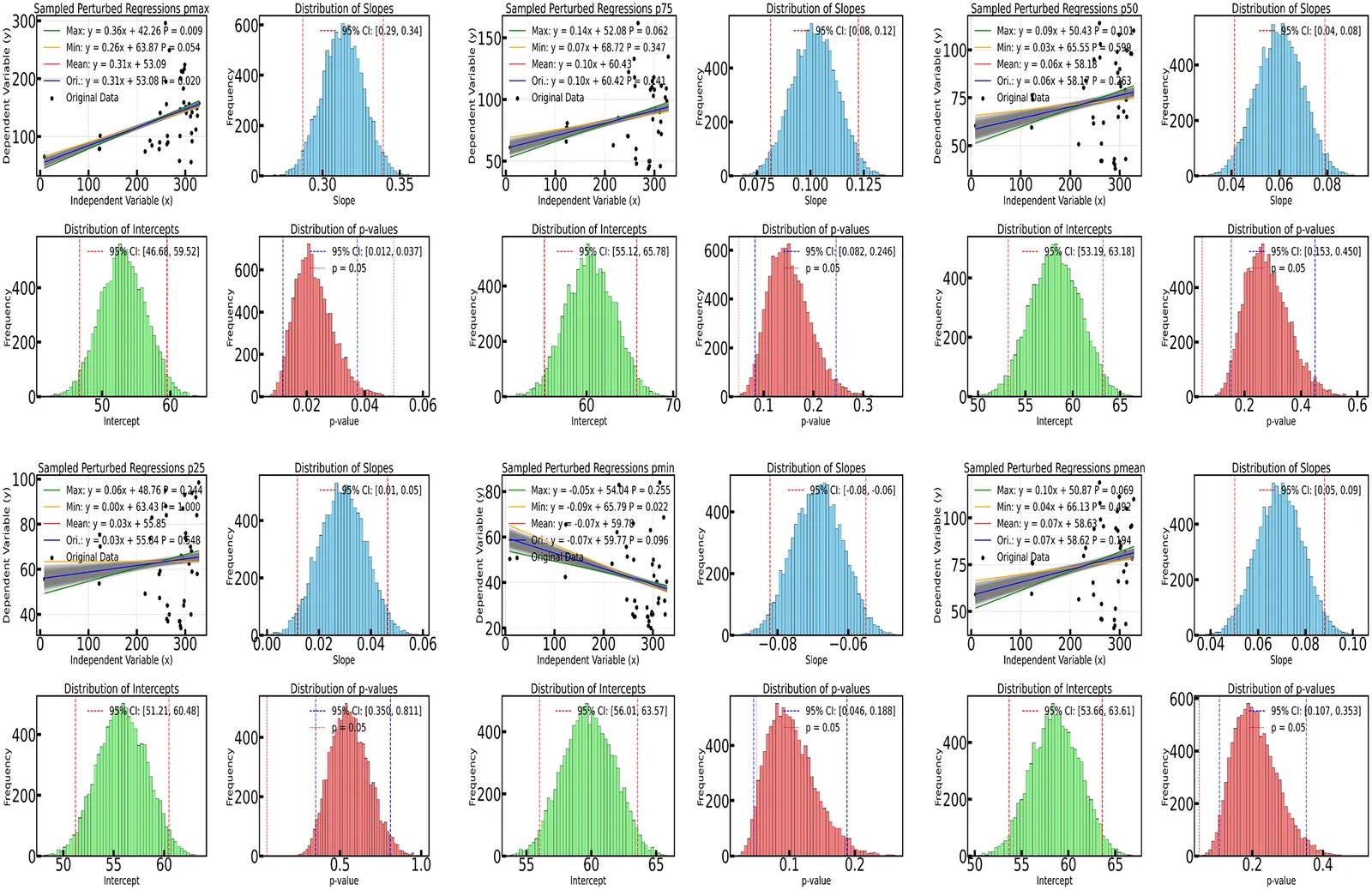
Time-series of long-term CO measurements and possible combinations of CO trends from linear regression analysis downwind of Asian emission areas and over the North Pacific upper troposphere, within longitudes $150^{\circ }E-140^{\circ }W$ and latitudes $25^{\circ }N-45^{\circ }N$. The time-series data and linear regression trends are stratified based on the statistical distributions of all data collected in a given month. Six statistical categories are presented in separate blocks of four panels: maximum values (top leftmost block), 75th percentile (top middle block), median (50th percentile) (top rightmost block), 25th percentile (bottom leftmost block), minimum values (bottom middle block), and mean values (bottom rightmost block). Each block of four panels provides a detailed breakdown of the CO trend analysis: Top Left Panel: Time-series data with all possible combinations of 10,000 linear regressions, including maximum, minimum, mean, and original regression equations, P-values from the original data; the original CO time-series data. Top Right Panel: Distribution of slopes from the 10,000 linear regression calculations. Bottom Left Panel: Distribution of intercepts from the 10,000 regression equations. Bottom Right Panel: Distribution of P-values from the 10,000 regression equations. Each distribution plot also displays the 95% confidence intervals (CI), providing a measure of statistical uncertainty.

Fig 6 presents time-series of long-term CO in-situ measurements from 1991 to 2012 in both the upper (blue) and lower (red) troposphere over the Asian emission region spanning longitudes $123^{\circ }E$ to $142^{\circ }W$ and latitudes $31^{\circ }N$ to $42^{\circ }N$. This region corresponds to the Northern Hemisphere warm conveyor belt (Fig 1(e)), through which heavy emissions from the Asian continent enter the North Pacific atmosphere (also see transport of Asian dust storm over the North Pacific [60]). The measurements are depicted as the maximum (upper left), 75th percentile (upper middle), 50th percentile (upper right), 25th percentile (lower left), minimum (lower middle), and mean (lower right) of all in-situ measurements in a given month. Linear regression analysis of the measurements reveals increasing trends in CO concentrations in both the lower and upper troposphere over the Asian emission regions.

**Fig 6:**
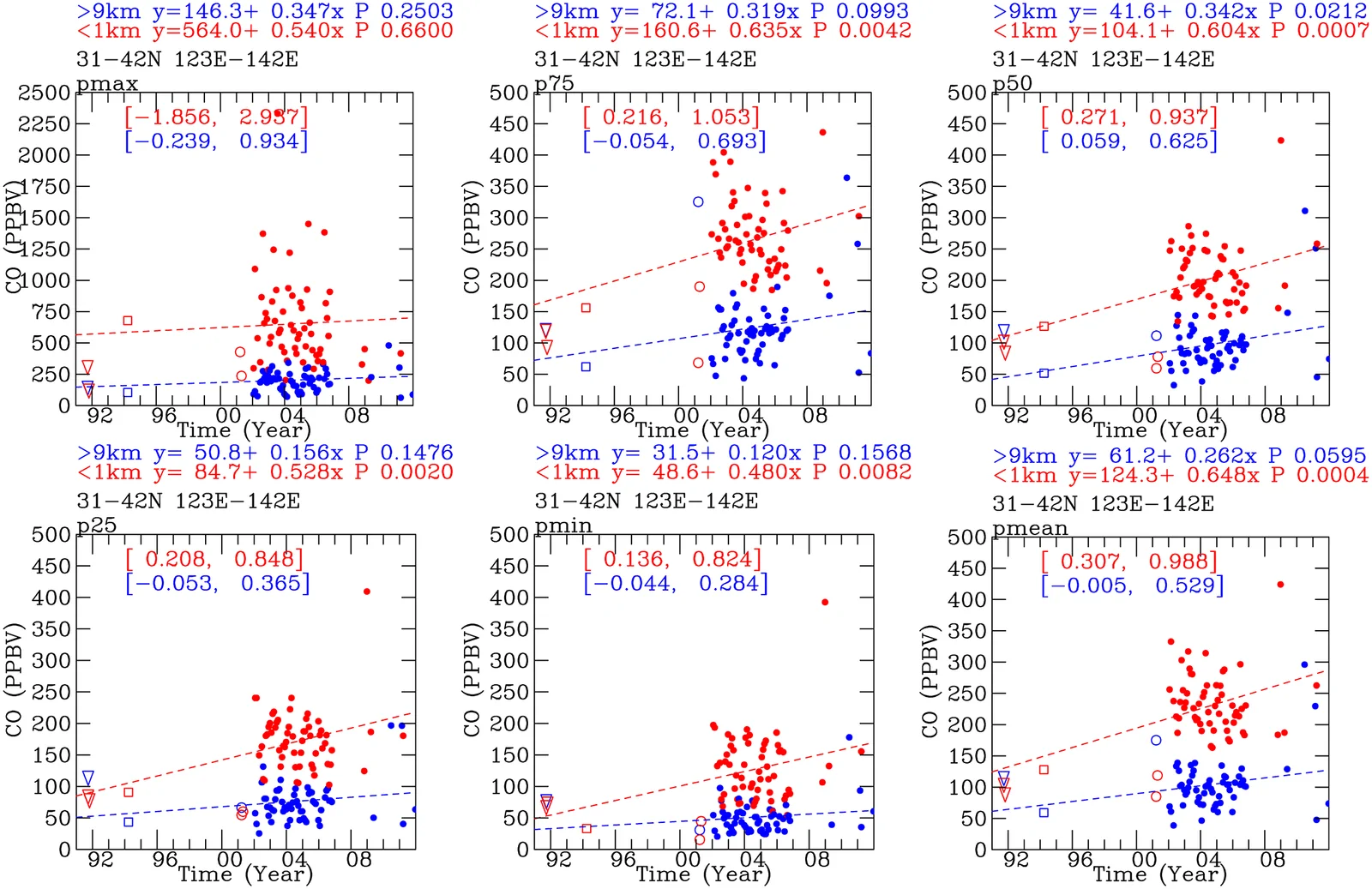
Time series of long-term CO in-situ measurements during 1991-2012 in the upper (blue) and the lower (red) troposphere over the Asian emission regions $123^{\circ }E-142^{\circ }W$, and latitudes $31^{\circ }N-42^{\circ }N$. The measurements are shown as the maximum (upper left), 75th (upper middle), 50th (upper right), 25th (lower left), minimum (lower middle), and the mean (lower right) of all in-situ measurements in a given month.

### Seasonal variability in the upper and lower troposphere

Fig 7 presents long-term measurements of CO, *O*_3_, and *H*_2_*O* over the Western Pacific (longitudes $123^{\circ }E-142^{\circ }W$, latitudes $31^{\circ }N-42^{\circ }N$) during the period 2000–2018. In comparison, Fig 8 shows the corresponding long-term measurements over Western Europe.

**Fig 7:**
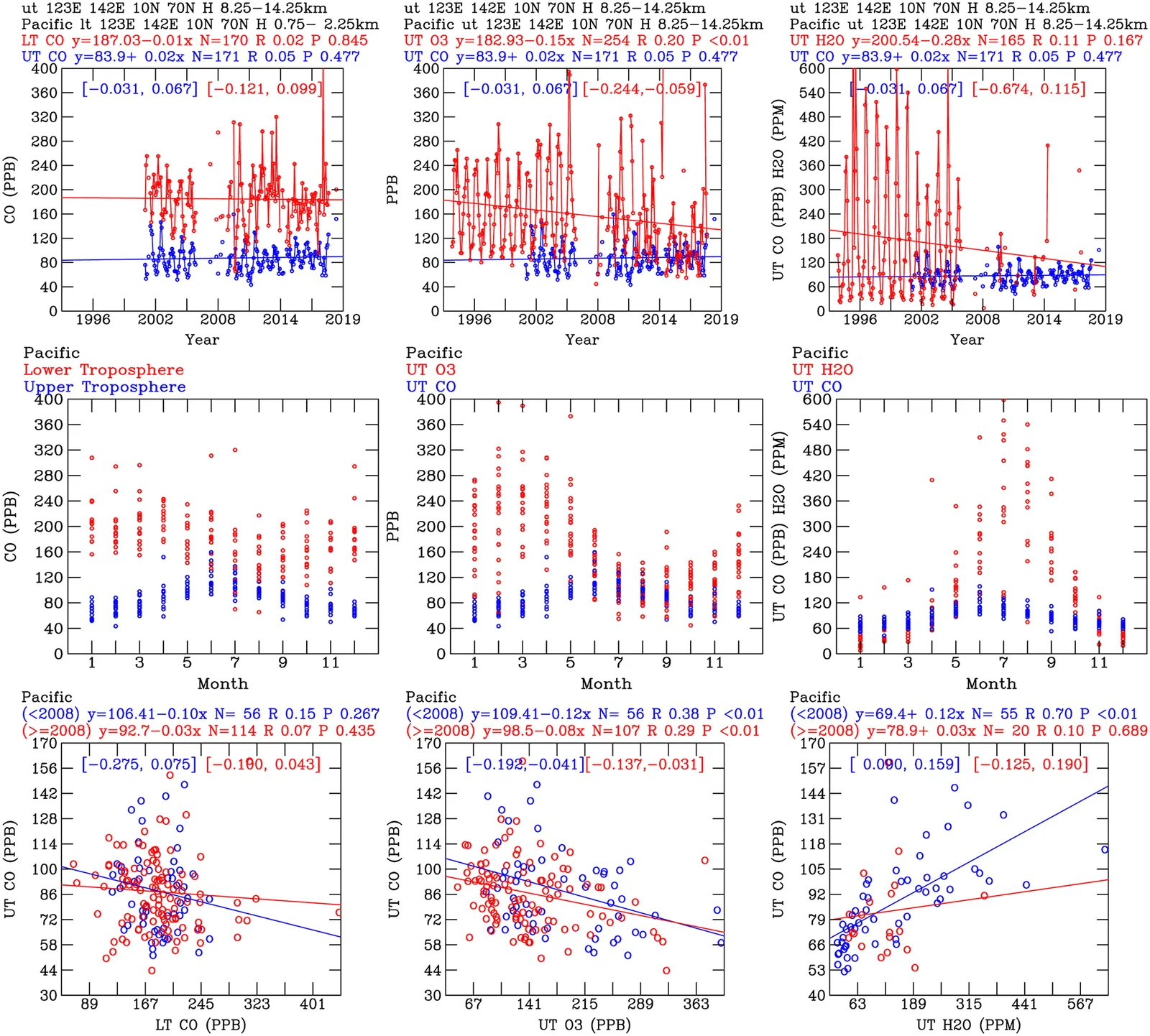
Long-term measurements are presented for: CO in the upper (blue) and lower (red) troposphere (top-left panel); *O*_3_ (blue) and CO (red) in the upper troposphere (top-middle panel); and CO (blue) and *H*_2_*O* (red) in the upper troposphere (top-right panel) during 2000–2018 over the Western Pacific (longitudes $123^{\circ }E$–$142^{\circ }W$, latitudes $31^{\circ }N$–$42^{\circ }N$). The middle row shows the monthly statistics of all measurements, while the bottom row compares the two observed variables. The 95% confidence intervals for each linear regression are indicated on each subplot.

**Fig 8:**
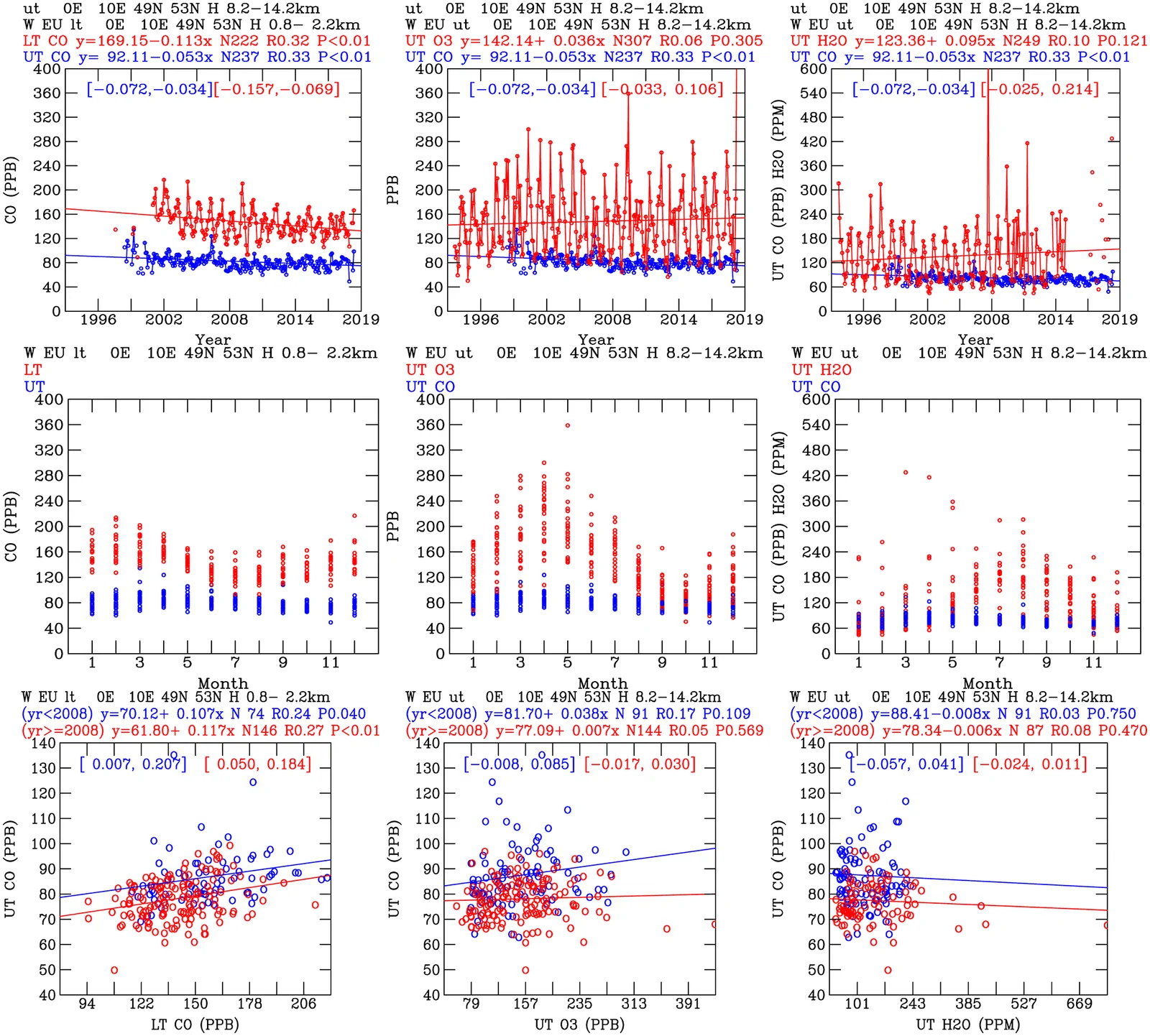
Long-term measurements are presented for: CO in the upper (blue) and lower (red) troposphere (top-left panel); *O*_3_ (blue) and CO (red) in the upper troposphere (top-middle panel); and CO (blue) and *H*_2_*O* (red) in the upper troposphere (top-right panel) during 2000–2018 over Western Europe (longitudes $0^{\circ }E$–$10^{\circ }E$, latitudes $49^{\circ }N$–$53^{\circ }N$). The middle row shows the monthly statistics of all measurements, while the bottom row compares the two observed variables. The 95% confidence intervals for each linear regression are indicated on each subplot.

In the Pacific region, the long-term trends of CO in the upper troposphere are positive, with 95% confidence intervals ranging from −0.031 to 0.067, indicating a tendency toward positive values despite some negative components. In the lower troposphere, the 95% confidence intervals extend from −0.121 to 0.099, suggesting a dominance of negative tendencies for CO. Although the p-values are high (0.477 in the upper troposphere and 0.845 in the lower troposphere), the distribution of the 95% confidence intervals reveals that positive tendencies dominate in the upper troposphere, while negative tendencies prevail in the lower troposphere.

Monthly statistics exhibit an out-of-phase behavior between CO concentrations in the upper and lower troposphere. During the summer months (June, July, and August), CO concentrations are higher in the upper troposphere and lower in the lower troposphere, whereas the opposite pattern is observed during the winter months (December, January, and February). This anti-correlation suggests that vertical pumping processes–-active during the summer when vertical convection is enhanced–-transport elevated CO from the lower to the upper troposphere, while reduced convection in winter results in lower CO levels aloft.

Evidence of this vertical pumping processes is also seen in the time-series measurements of CO and *O*_3_ in the upper troposphere. Here, CO exhibits positive trends, whereas *O*_3_ trends are negative. The monthly CO values are higher in summer than in winter, while *O*_3_ values peak in spring rather than summer. The negative *O*_3_ trends imply a reduced downward intrusion of elevated stratospheric *O*_3_ and enhanced pumping of low-*O*_3_ air from the lower troposphere, leading to a negative correlation between *O*_3_ and CO in the upper troposphere.

Analysis of *O*_3_ in the upper troposphere shows negative trends, with 95% confidence intervals between −0.244 and −0.059. The entirely negative intervals and a p-value below 0.01 indicate a statistically significant decrease in *O*_3_ over the Pacific. Moreover, the anti-correlation between CO and *O*_3_–-with high CO associated with low *O*_3_ and vice versa–-further corroborates the vertical pumping process, as lower-tropospheric air (rich in CO) is lifted into the upper troposphere where *O*_3_ is relatively lower.

Fig 7 also compares CO and *H*_2_*O* in the upper troposphere. Here, the trends for *H*_2_*O* are negative, whereas the trends for CO remain positive. Seasonal variations reveal that *H*_2_*O* concentrations are higher during summer months, coinciding with elevated CO levels, resulting in a positive correlation between *H*_2_*O* and CO. For the period before 2008, the 95% confidence intervals for the *H*_2_*O*–CO correlation range from 0.090 to 0.159 and are statistically significant (*p* < 0.01). For the period after 2008, the intervals range from −0.125 to 0.190, although the positive correlations continue to dominate.

In summary, over the North Pacific, CO in the upper troposphere is negatively correlated with CO in the lower troposphere and with *O*_3_ in the upper troposphere, while it is positively correlated with *H*_2_*O* from the lower troposphere. These relationships support the role of vertical pumping in redistributing CO, *H*_2_*O*, and *O*_3_ throughout the troposphere, particularly during the active convective periods of summer.

Fig 8 compares the time-series of CO, *O*_3_, and *H*_2_*O* in the upper troposphere over the downwind region of the North Atlantic in western Europe (longitude: $0^{\circ }E$–$10^{\circ }E$, latitude: $49^{\circ }N$–$53^{\circ }N$). In this region, the time-series trends for CO are statistically significant and negative in both the upper troposphere (95% confidence intervals between −0.072 and −0.034) and the lower troposphere (95% confidence intervals between −0.157 and −0.069), which is consistent with reduced CO emissions. In contrast, the trends for *H*_2_*O* (95% confidence intervals between −0.025 and 0.214) and *O*_3_ (95% confidence intervals between −0.033 and 0.106) are dominated by positive values.

CO concentrations show statistically significant positive correlations between the upper and lower troposphere. For data before 2008, the 95% confidence intervals for these correlations range from 0.007 to 0.207, and for data after 2008 they range from 0.050 to 0.184. CO levels are elevated during the winter months when baroclinic waves and the associated warm conveyor belt processes are active in transporting CO from the lower to the upper troposphere.

The positive trends in upper tropospheric *O*_3_ over the North Atlantic during 1994–2018, as observed in IAGOS data, reflect contributions from both stratospheric and anthropogenic sources. Enhanced stratosphere-to-troposphere exchange (STE), associated with intense wave activity and active baroclinic dynamics, introduces ozone-rich air from the lower stratosphere into the upper troposphere, particularly during winter and spring [78]. Additionally, long-range transport of ozone and its precursors (e.g., NOx, CO) from Asia significantly contributes to these trends. Gaudel et al. [68] report that increasing Asian emissions, particularly from China and India, have driven upper tropospheric ozone increases across the Northern Hemisphere, with the North Atlantic experiencing modest but significant trends of 1.5–4.5 ppbv per decade. Wang et al. [34] further attribute these trends to the equatorward redistribution of anthropogenic emissions, with Asian NOx emissions enhancing ozone production during transatlantic transport.

In the upper troposphere, CO and *O*_3_ exhibit a positive correlation, with 95% confidence intervals ranging from −0.008 to 0.085 before 2008 and from −0.017 to 0.030 after 2008, reflecting the influence of both stratospheric intrusions and anthropogenically influenced air masses. In contrast, correlations between CO and *H*_2_*O* are predominantly negative, with 95% confidence intervals between −0.057 and 0.041 before 2008 and between −0.024 and 0.011 after 2008. These patterns indicate that higher CO is associated with higher *O*_3_ and lower *H*_2_*O*, characteristic of dry, ozone-rich air from the lower stratosphere or polluted air masses transported from Asia. Conversely, lower CO corresponds to lower *O*_3_ and higher *H*_2_*O*, suggestive of moist, tropospheric air uplifted via warm conveyor belt (WCB) processes over the North Atlantic. These characteristics highlight the interplay of vertical downward transport from the stratosphere, long-range transport of Asian emissions, and upward transport via WCBs, shaping the observed O3 trends and chemical signatures in the North Atlantic’s upper troposphere.

The vertical pumping processes discussed above corroborate the CO tape recorder process [79]. Schoeberl et al. [79] explain that, despite CO’s relatively short chemical lifetime, its vertical profile in the tropical upper troposphere and lower stratosphere retains a clear imprint of the seasonal variations at its source. In their analysis of the “CO tape recorder,” they emphasize that the seasonal cycle is strongly influenced by changes in tropospheric CO–-much of which is attributed to biomass burning–-being convectively injected into the upper troposphere. The key to the tape recorder effect lies in the balance between CO’s chemical loss and the rate of vertical transport. In the tropics, strong convective processes rapidly lift CO from the lower troposphere into the upper troposphere and lower stratosphere on a timescale that is comparable to or even shorter than its chemical lifetime. As a result, the seasonal variations in CO concentrations are effectively “recorded” in the vertical profile, even as chemical removal processes continue to act.

### Variability of CO profiles from 2012 to 2018

The previous section demonstrated evidence of the vertical pumping of CO and *H*_2_*O* from the lower troposphere to the upper troposphere, which plays a crucial role in the vertical redistribution of chemicals from ground-level sources throughout the troposphere. We note that, while horizontal transport is indeed a key process in the overall dispersion of atmospheric chemicals, our focus in this section is on the vertical exchange processes. The evidence presented–-including seasonal variability in vertical profiles and the distinct “tape recorder” signal–-strongly supports the role of vertical pumping (e.g., convective lifting and large-scale overturning) in redistributing CO and *H*_2_*O* from near the surface to the upper troposphere. Although horizontal transport (e.g., advection by synoptic-scale winds) also influences the spatial distribution of pollutants, its effects are more dominant in the lateral spreading rather than in the vertical stratification of trace gases.

In this section, we examine the vertical profiles of CO over various regions, as shown in Fig 1, using in-situ measurements collected by the IAGOS aircraft.

Fig 9 presents annual profiles of in-situ CO measurements from 2012 to 2018. CO concentrations near the surface range between 160 ppbv and 560 ppbv, while concentrations in the upper troposphere vary between 40 ppbv and 120 ppbv. Significant variations in CO were observed at altitudes lower than 3 km and higher than 9 km. A sharp decrease in CO concentration was observed vertically, from near the surface to about 5 km, followed by a gradual reduction from 5 km to 12 km. Fig 9 illustrates that CO is produced from surface emissions and transported upward through vertical pumping processes. We note that CO concentrations in the upper troposphere over the North Pacific (Fig 9(a)) and the warm conveyor belt (Fig 9(b)) exhibit more variability than in other regions during the 2012–2018 period.

**Fig 9:**
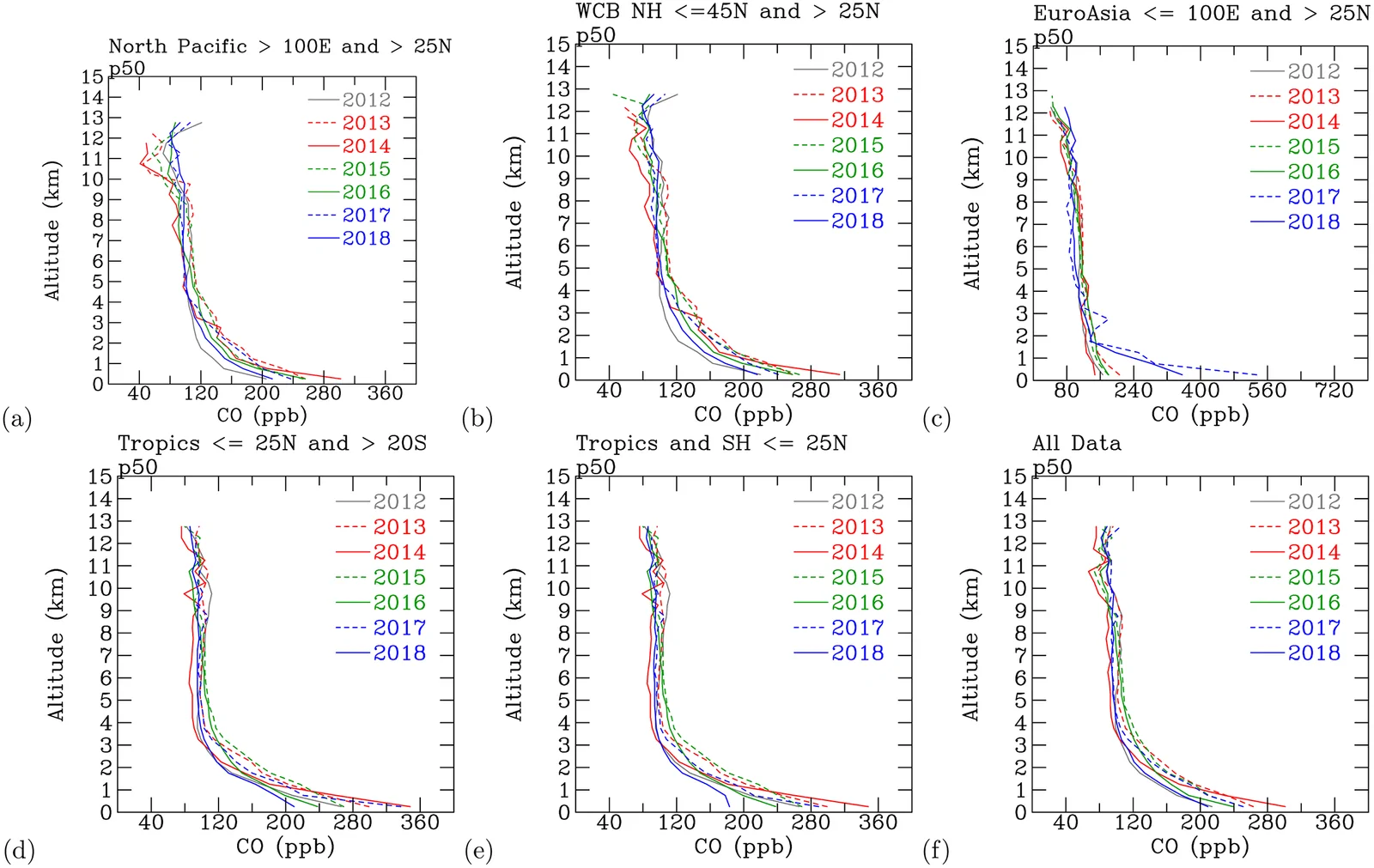
Annual profiles of in-situ CO measurements during the period 2012-2018. Profiles are shown for the 50th percentile of measurement data: (a) downstream of heavy Asian emission areas over north Pacific, in longitudes east of $100^{\circ }E$ and latitudes north of $25^{\circ }N$; (b) Northern Hemisphere warm conveyor belt, in latitudes $25^{\circ }N-45^{\circ }N$; (c) upstream of Asian emission areas over Euro-Asia regions, in longitudes west of $100^{\circ }E$ and latitudes north of $25^{\circ }N$; (d) tropical regions, in latitudes $25^{\circ }N-20^{\circ }S$; (e) tropical regions and Southern Hemisphere, in latitudes south of $25^{\circ }N$; and (f) all data.

Fig 10 presents profiles of CO trends for the period 2012–2018 derived from in-situ measurements, with results shown for different percentiles and the mean. For lower troposphere trends, negative monthly mean CO trends align with the mean and 50th percentile trends between 3 and 4 km, as observed by IAGOS from 2012–2018. Airplane measurements indicate positive trends over East Asia and the North Pacific, while negative trends are found over the North Atlantic–-consistent with a steady decline in technological emissions estimated by EDGARv6.1.

**Fig 10:**
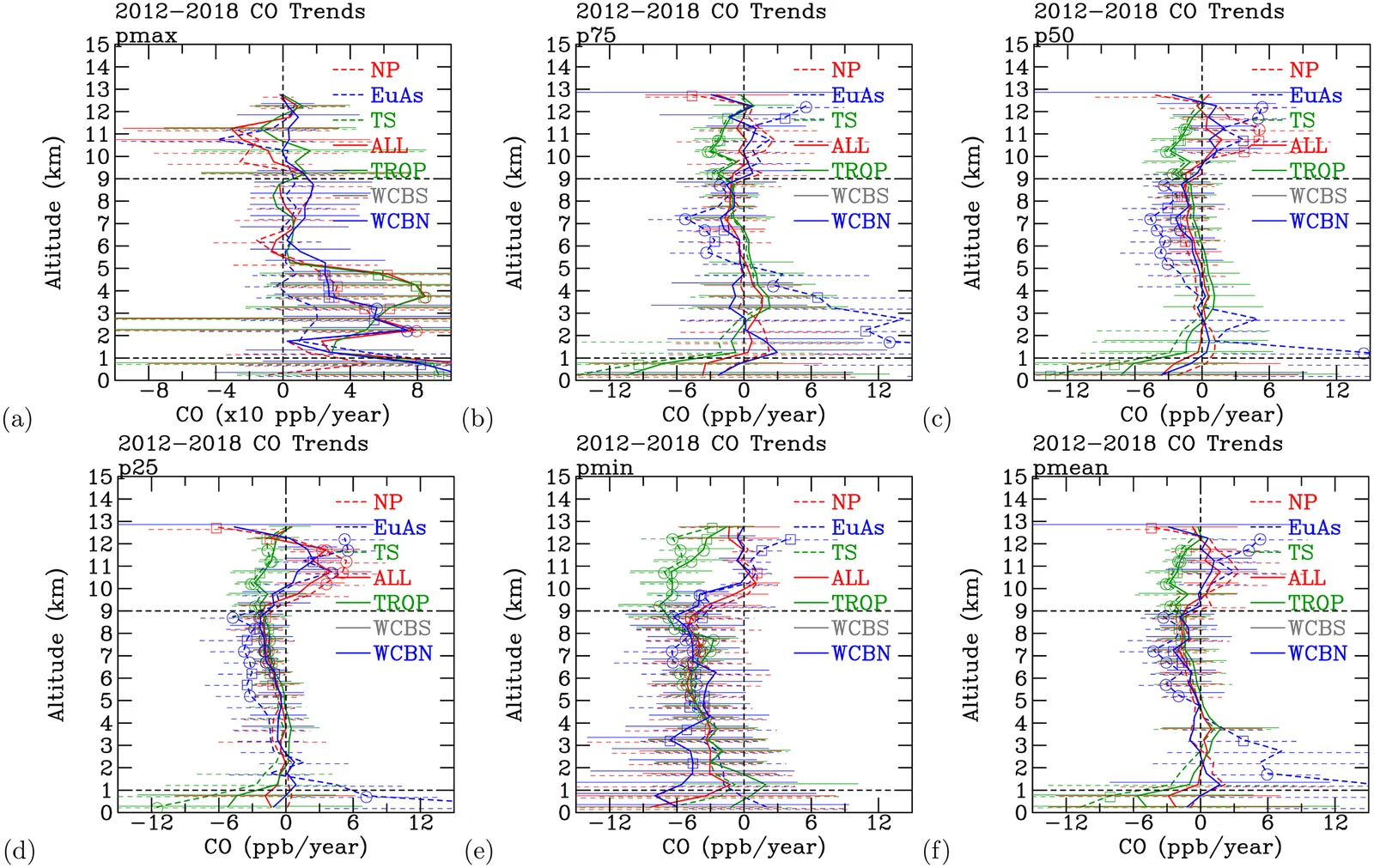
Profiles of CO trends for the period 2012-2018. The trends were shown for regions downwind of the east Asia emission areas over the North Pacific region (NP; dashed red), upwind of the east Asia emission areas over the Euro-Asia region (EuAS; dashed blue), tropical regions ($23.5^{\circ }N-23.5^{\circ }S$) and the Southern Hemisphere (TS; green), Southern Hemisphere warm conveyor belt (WCBS; gray), Northern Hemisphere warm conveyor belt (WCBN; blue), and all regions (ALL; red). The trends are shown for the (a) maximum, (b) 75th percentile, (c) 50th percentile, (d) 25th percentile, (e) minimum, and (f) the mean of the in-situ measurement data. The 95% confidence intervals for the linear trend estimates at each altitude and for each region are plotted alongside the trend lines to assess the statistical significance of the trends.

For CO tendencies, when averaged over the regions shown in Fig 9, the maximum CO displays distinctive positive trends in the troposphere (especially below 6 km), whereas the minimum CO trends are negative below 10 km, reflecting background CO levels. Positive trends suggest that chemical sources (including the conversion of hydrocarbons to CO and direct CO emissions) are dominant at the highest concentrations, while negative trends imply that chemical sinks are primarily responsible for CO removal.

For the percentile comparisons, trends for the 75th percentile CO resemble those for the maximum CO, with the most significant positive trends occurring below 6 km. In contrast, the 25th percentile and minimum CO trends are generally negative in the troposphere, indicating that chemical sinks dominate below 9 km, while chemical sources are relatively more influential in the upper troposphere. The 50th percentile CO trends mirror the mean trends–-showing negative values below 9 km (except for slight positive tendencies between 3 and 5 km) and positive trends above 9 km. Chemical Processes: Positive tendencies below 5 km suggest strong contributions from chemical sources, whereas positive tendencies above 9 km likely result from reduced chemical sinks. The consistently negative trends observed in the minimum CO profile indicate robust removal of CO throughout most altitudes.

For the role of error bars, the vertical distribution of the error bars closely follows the pattern of the linear trends for each region. When the trends are negative, the error bars are predominantly negative; conversely, when trends are positive, the error bars are skewed toward positive values. This inclusion reinforces the characteristic distribution of the linear trends.

We note that are is a significant variability (and large error bars) in Fig 10. For variability in error bars, larger variations in error bar ranges are mostly seen at lower altitudes, reflecting the direct impact of measurements near industrial and CO emission areas. For example, the maximum CO (pmax) shows wide error bar ranges, consistent with variability associated with elevated CO transported from heavy emission areas (as illustrated by the CO tape recorder effect from biomass burning in Schoeberl et al. [79]). In contrast, the error bars for the minimum (pmin) and the 25th percentile CO are more confined around their central trend values, while the mean and 50th percentile trends (except near the extremes, below 3 km and above 13 km) are similarly well constrained.

### What controls long-term CO trends in the atmosphere

#### IMS simulations and verifications against MLO, IAGOS, MOPITT measurements.

Fig 11 presents time-series simulation results from the IMS model, along with a comparison with Mauna Loa measurements. The IMS simulation results are depicted for both the lower troposphere (red) and the upper troposphere (blue) in Fig 11(a), while the Mauna Loa measurements are represented in black. The 95% confidence intervals of the CO trends are shown on the plot. For the lower troposphere, the 95% confidence intervals of the CO trends from the Mauna Loa measurements are between −0.077 and 0.050, dominated by the negative CO trends. The 95% confidence intervals of the CO trends from the IMS model are between −0.055 and 0.016, also dominated by the negative CO trends. The confidence intervals of the model trends are within the ranges of confidence intervals from the observation. The modelled 95% confidence intervals of the CO trends in the upper troposphere are between −0.043 and 0.022, also dominated by the negative CO trends. The negative CO trends over the Mauna Loa upper troposphere are consistent with the CO negative trends over the tropical regions from IAGOS observations, as shown in Fig 10 (in green line of TROP).

**Fig 11:**
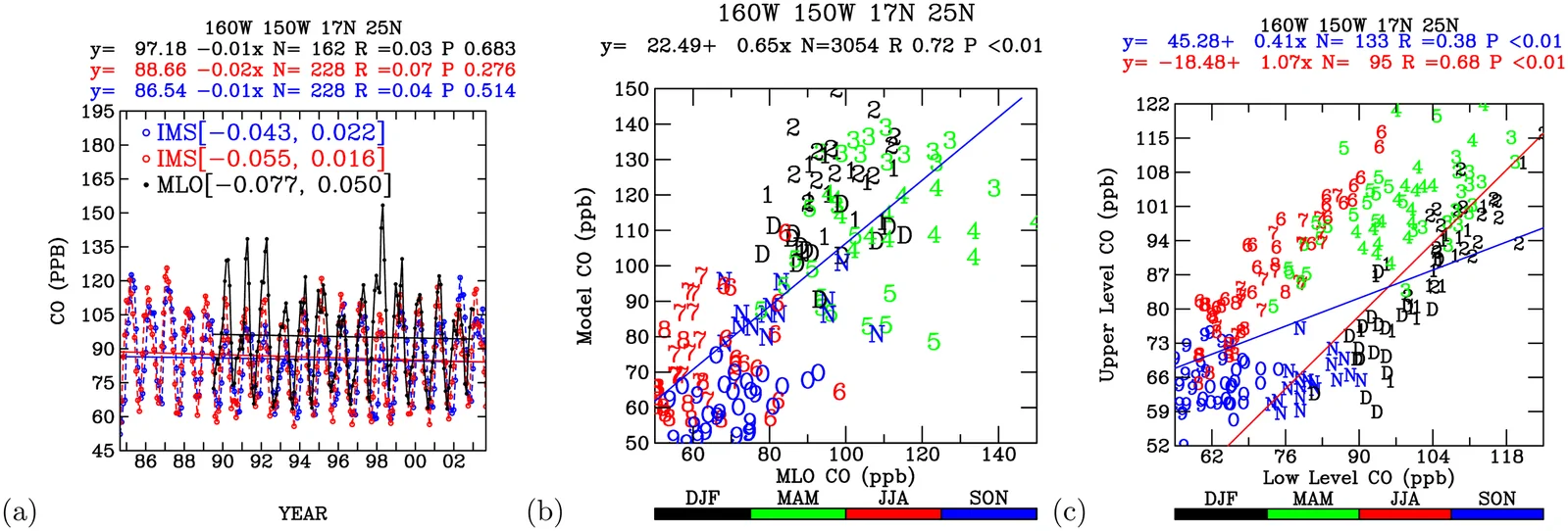
Time-series simulation results from the IMS model and the comparison with the Mauna Loa measurements. (a) Time-series CO from the IMS simulation, where red is for the lower troposphere, and blue is for the upper troposphere. The 95% confidence intervals of the CO trends are shown on the plot. The Mauna Loa measurements are shown in black. (b) Scatter plot analysis of the IMS simulated CO versus the Mauna Loa measurements of CO. The colors indicate the month of each data point. (c) Scatter plot analysis of the IMS simulated CO in the upper troposphere versus the IMS simulated CO in the lower troposphere. Positive correlation (blue linear regression line) between the upper and the lower troposphere CO during the summer (JJA; June, July, and August) to the autumn (SON; September, October, and November). Positive correlation (red linear regression line) from winter (DJF; December, January, February) to spring (MAM; March, April, May) months.

In Fig 11(b), a scatter plot analysis of the IMS simulated CO versus the Mauna Loa measurements of CO is shown. The comparison reveals a strong correlation between the IMS simulations and the Mauna Loa measurements, although the IMS model tends to overestimate CO compared to the Mauna Loa measurements. Fig 11(c) illustrates a scatter plot analysis of the IMS simulated CO in the upper troposphere versus the IMS simulated CO in the lower troposphere, with colors indicating the months of the data.

During the summer to autumn months, modeled CO concentrations in the upper troposphere are positively correlated with those in the lower troposphere. These months coincide with the period of intense Asian summer monsoon over the North Pacific. During the winter to spring months, modeled CO concentrations in the upper troposphere show a positive correlation with those in the lower troposphere. These months correspond to the period of intense Asian winter monsoon, characterized by frontal activity associated with the development of baroclinic waves.

Fig 12 presents the time-series simulation results from the IMS model (blue), compared against IAGOS measurements (sky blue) and the linear regression prediction from IMS (1984–2003) projected to the end of 2020. The 95% confidence intervals of the trend estimates are indicated in brackets on each subplot. The figure is structured into two major regions of the upper troposphere for comparisons: over the North Atlantic and over the North Pacific.

**Fig 12:**
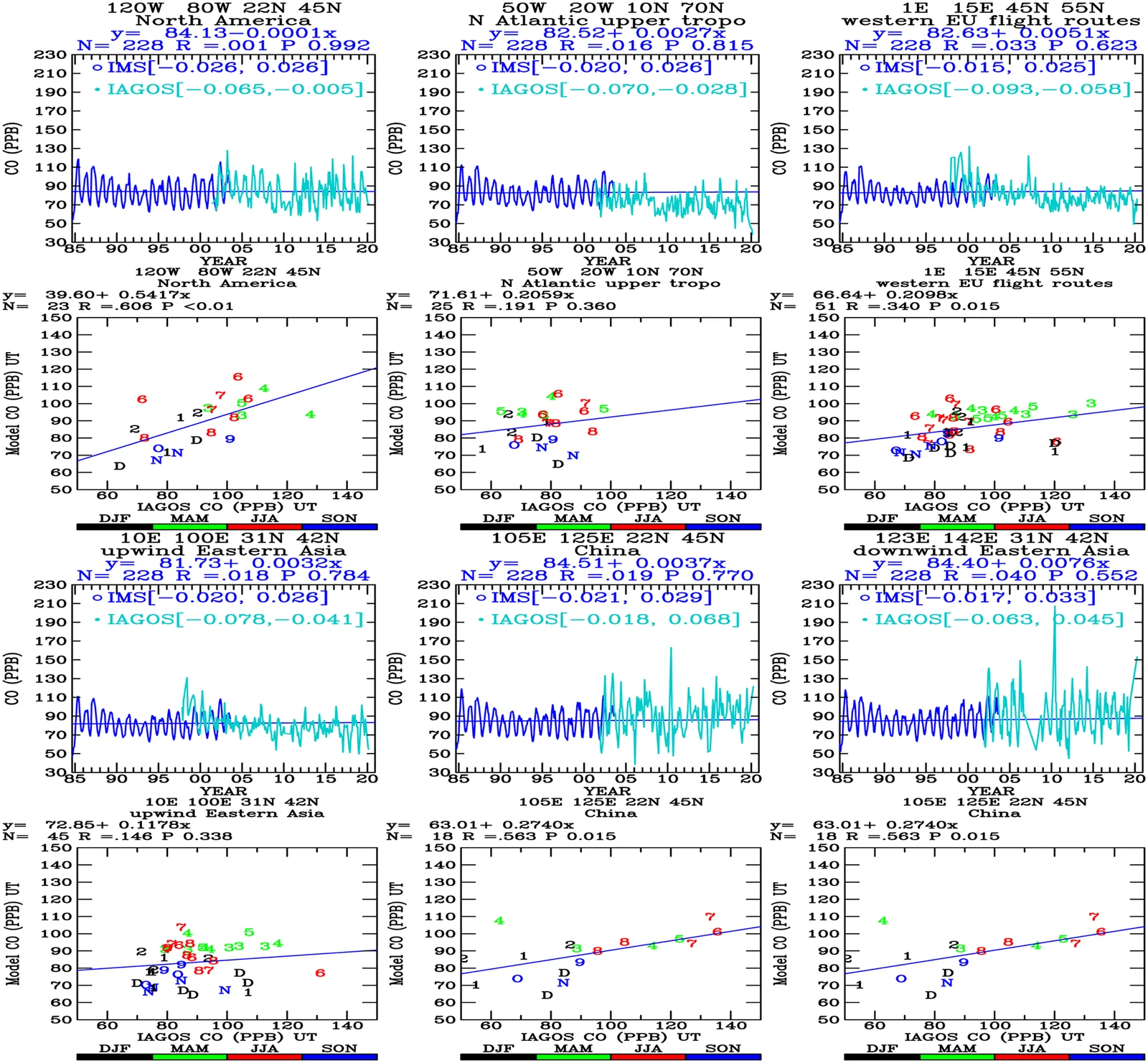
Time-series simulation results from the IMS model (blue), the comparisons with the IAGOS measurements (sky blue), and the linear regression prediction from the IMS 1984-2003 simulation to the end of 2020. The 95% confidence intervals of the trends are shown within the brackets on each time-series subplot: Over the upper troposphere of the North Atlantic (top-left panel, upstream of North American; middle top panel, North Atlantic; and top-right panel, downstream, western Europe); and over the North Pacific (third panel left, upstream of China; third middle panel, China. third right panel, downstream of China). The second and the bottom panel shows corresponding scatted analysis from the top and the third panel.

For the North Atlantic upper troposphere (top row), the top-left panel shows CO trends upstream of North America $\left(120^{\circ }W-80^{\circ }W,25^{\circ }N-45^{\circ }N\right)$; the Middle-top panel shows CO trends over the North Atlantic $\left(80^{\circ }W-10^{\circ }W,10^{\circ }N-70^{\circ }N\right)$; the top-right panel shows CO trends downstream over Western Europe $\left(10^{\circ }E-45^{\circ }E,45^{\circ }N-55^{\circ }N\right)$.

For the North Pacific upper troposphere (third row), the third-left panel shows CO trends upstream of China $\left(120^{\circ }E-80^{\circ }E,25^{\circ }N-45^{\circ }N\right)$; the middle-third panel shows CO trends over China $\left(80^{\circ }E-150^{\circ }E,25^{\circ }N-45^{\circ }N\right)$; the third-right panel shows CO trends downstream of China over the Pacific $\left(150^{\circ }E-160^{\circ }W,25^{\circ }N-45^{\circ }N\right)$.

For statistically significant trends of the 95% confidence intervals, the IMS model shows long-term trends of CO are between −0.026 and 0.025, while the observed long-term trends of CO from IAGOS are all negative (between −0.093 and −0.058). The IMS model were calculated with fixed emissions, while the observed negative CO trends were consistent with significant reductions of CO emissions over the North America and western Europe (Fig 1(f)). Hence, the all negative trends from the IAGOS observations reflect the effects of the CO emission reductions.

For over the North Pacific, the IMS models shows CO trends between −0.021 and 0.033, while the observed trends from IAGOS observations are between −0.078 (upstream of the North Pacific) and 0.033 (downstream of the North Pacific). The positive CO trends downstream of the North Pacific are consistent with the model trends, consistent with increase in CO emissions over the North Pacific industrial areas (Fig 1(f)). The confidence intervals confirm the significance of positive or negative trends in specific regions.

These comparisons yield significant findings. Notably, the IMS 1984–2003 simulations were conducted with constant technological CO and emissions from hydrocarbons, while the IAGOS measurements reflect the real CO and hydrocarbon emissions during 2004–2021. Variations in technological CO emissions are estimated in Fig 1(f). Over the North Atlantic upper troposphere, IMS predictions exceed the IAGOS measurements. This suggests a significant reduction in CO chemical sources, including CO and hydrocarbon emissions, and an increase in upper troposphere/lower stratosphere (UTLS) air exchange processes transporting low CO from the lower stratosphere to the upper troposphere. An increase in UTLS air exchange processes can be driven by several factors. For example, enhanced deep convection–-particularly in the tropics–-can rapidly transport air from the lower troposphere into the UTLS. In addition, increased planetary wave activity in the extratropics can lead to more frequent tropopause folds, which facilitate the exchange of air between the stratosphere and troposphere. Changes in large-scale circulation patterns, such as a strengthened Brewer–Dobson circulation, may also contribute to more vigorous UTLS exchange processes. The efficiency with which CO reaches the stratosphere by this route is set by the competition between its chemical lifetime and the timescale of the transport. CO is removed almost entirely by reaction with the hydroxyl radical, giving a tropospheric lifetime of roughly one to three months: closer to one month in the warm, humid, OH-rich tropical lower troposphere, and extending to several months in the cold, dry upper troposphere and at high latitudes in winter, where OH abundances are low. Averaged globally the lifetime is approximately two months, which is short compared with the interhemispheric mixing time but long compared with the timescale of deep convection. This ordering is what makes CO informative here. Convective lifting in the tropics delivers boundary-layer air to the tropopause in a day or less, far faster than chemical loss, so the seasonal signature of surface emissions is preserved as the air enters the lower stratosphere – the basis of the CO tape recorder discussed above [79]. The subsequent ascent of that air within the rising branch of the Brewer–Dobson circulation, however, takes several months to more than a year, which is long compared with the CO lifetime. The Brewer–Dobson circulation therefore transmits a strongly attenuated CO signal into the middle stratosphere: it controls the rate at which tropospheric air is supplied to the stratosphere, but most of the CO carried in that air is oxidised to *CO*_2_ during ascent. A strengthening of the circulation consequently affects upper-tropospheric CO mainly through the compensating downward branch, which returns CO-poor stratospheric air to the extratropical upper troposphere, rather than by exporting appreciable amounts of CO upward. A detailed discussion of these mechanisms is provided by Holton et al. [80], which reviews how both convective and synoptic-scale dynamics play critical roles in driving stratosphere–troposphere exchange processes.

Over the North Pacific upper troposphere and downwind of the East Asia emission areas, IMS 2004–2020 predictions fall below the IAGOS 2004–2020 measurements. This indicates that the impacts of continuous CO emissions during 2004–2020 outweigh the effects of the constant CO emissions used in the 1984–2003 simulations. Over the upper troposphere upwind of the East Asia emission areas, IMS 2004–2020 predictions approximate the 2004–2020 in-situ measurements. This suggests reductions in chemical sources and emissions of CO and hydrocarbons upwind of the East Asia emission areas.

For the comparison between the model and the IAGOS Observations, the second and bottom rows display scatter analysis corresponding to the top and third-row time-series panels, respectively. These scatter plots show the relationships between observed and simulated CO values, supporting the validation of IMS model trends against IAGOS observations. The IMS simulation aligns well with IAGOS data, validating the robustness of modeled CO variability. For the upstream versus downstream patterns, CO concentrations increase or decrease depending on proximity to major anthropogenic sources (e.g., China, North America, Europe). The scatter plots demonstrate that, the IMS simulations are positively correlated with the IAGOS measurements. However, the model underestimates elevated CO while it overestimate low CO compared with the IAGOS in-situ measurements, consistent with findings from previous studies [25].

Fig 13 illustrates time-series simulation results from the IMS model alongside comparisons with MOPITT measurements. The IMS simulations depict the upper troposphere (in blue) and the lower troposphere (in red) for (a) the North Atlantic ($50^{\circ }W-20^{\circ }W,10^{\circ }N-70^{\circ }N$; downwind of North American emission sources), and (e) the North Pacific ($150^{\circ }E-140^{\circ }W,25^{\circ }N-45^{\circ }N$; downwind of Asian emission sources). MOPITT measurements are represented for the lower troposphere (in light green) and the upper troposphere (in sky blue).

**Fig 13:**
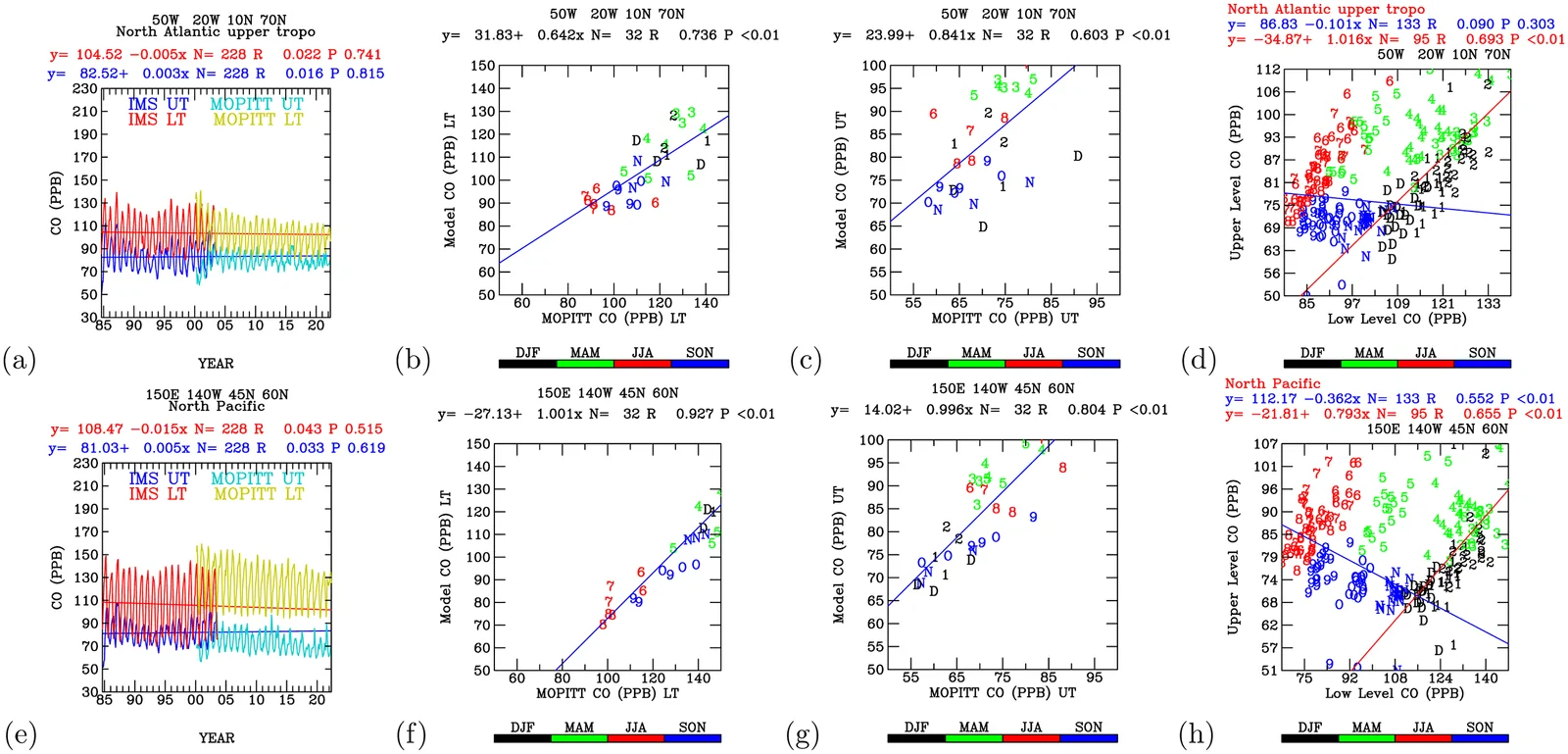
Time-series simulation results from the IMS model and the comparisons with the MOPITT measurements. The IMS simulations are shown for the upper troposphere (blue) and the lower troposphere (red) for (a) the North Atlantic ($50^{\circ }W-20^{\circ }W,10^{\circ }N-70^{\circ }N$; downwind of the North America emission sources), and (e) the North Pacific ($150^{\circ }E-140^{\circ }W,25^{\circ }N-45^{\circ }N$; downwind of the Asia emission sources). The MOPITT measurements are shown for the lower troposphere (light green) and the upper troposphere (sky blue). The IMS model compared with the MOPITT measurements for the lower troposphere over (b) the Atlantic and (f) the Pacific; and for the upper over (c) the Atlantic and (g) the Pacific when the time of measurements are overlapped. The model CO in the upper troposphere correlated with the CO in the lower troposphere over the (d) Atlantic and (h) the Pacific. The color and digits indicate the month of the data. The linear anti-correlation lines (blue) are shown for the summer (JJA) to autumn (SON) months. The linear positive correlation lines (red) are shown for the winter (DJF) to spring (MAM) months.

Comparison between the IMS model and MOPITT measurements is presented for the lower troposphere over (b) the Atlantic and (f) the Pacific, and for the upper troposphere over (c) the Atlantic and (g) the Pacific. Additionally, the correlation between model CO in the upper troposphere and CO in the lower troposphere is depicted over the (d) Atlantic and (h) Pacific. The color coding and numerical annotations indicate the month of the data.

The IMS model exhibits strong correlation with MOPITT measurements across both the lower and upper troposphere in the North Atlantic and the North Pacific. Notably, MOPITT estimates tend to be higher than IMS results over the North Pacific (Fig 13(b)) and the North Atlantic (Fig 13(f)). Conversely, for the upper troposphere, MOPITT estimates appear lower than IMS results over the North Pacific (Fig 13(c)) and the North Atlantic (Fig 13(g)).

During the summer (JJA) to autumn (SON) and winter (DJF) transition months, IMS CO levels in the upper troposphere exhibit negative correlations with IMS CO in the lower troposphere over the North Pacific (Fig 13(d)) and North Atlantic (Fig 13(h)). This negative correlation is particularly evident during the SON months, consistent with seasonal patterns observed in IAGOS in-situ measurements over the North Pacific (Figure 7). Such negative correlations are indicative of convective vertical transport processes that pump CO from the lower to the upper troposphere. We note that this signal is more robust over the North Pacific than over the North Atlantic, reflecting stronger convective activity over the Asian continent compared to North America. In contrast, during the winter (DJF) to spring (MAM) and summer (JJA) months, CO levels in the upper troposphere show positive correlations with those in the lower troposphere, reflecting the seasonal build-up of CO in the lower troposphere and subsequent transport aloft by warm conveyor belts.

The IMS model and MOPITT measurements are sources capable of providing CO distribution on a global scale. The analysis confirms the consistency between IMS model outputs and MOPITT measurements, reinforcing the observation of CO transport from the lower to the upper troposphere during summer months in the Northern Hemisphere, as evidenced by IAGOS in-situ measurements over the North Pacific. Further validation of MOPITT measurements with Mauna Loa in-situ data is planned for the next phase.

Fig 14 illustrates time-series CO measurements from MOPITT and their comparison with Mauna Loa measurements: (a) The MOPITT CO measurements during the period 2000–2022 over the upper (blue) and the lower (red) troposphere in an area containing Mauna Loa ($160^{\circ }W$-$150^{\circ }W$, $17^{\circ }N$-$25^{\circ }N$). The Mauna Loa measurements are shown for the period 1989–2022 (black). Also depicted are the linear regressions of the MOPITT data over the lower and the upper troposphere, respectively, and the Mauna Loa data. (b) The MOPITT data over the lower troposphere correlated with the Mauna Loa measurements. The colors and the digits indicate the months of the data.

**Fig 14:**
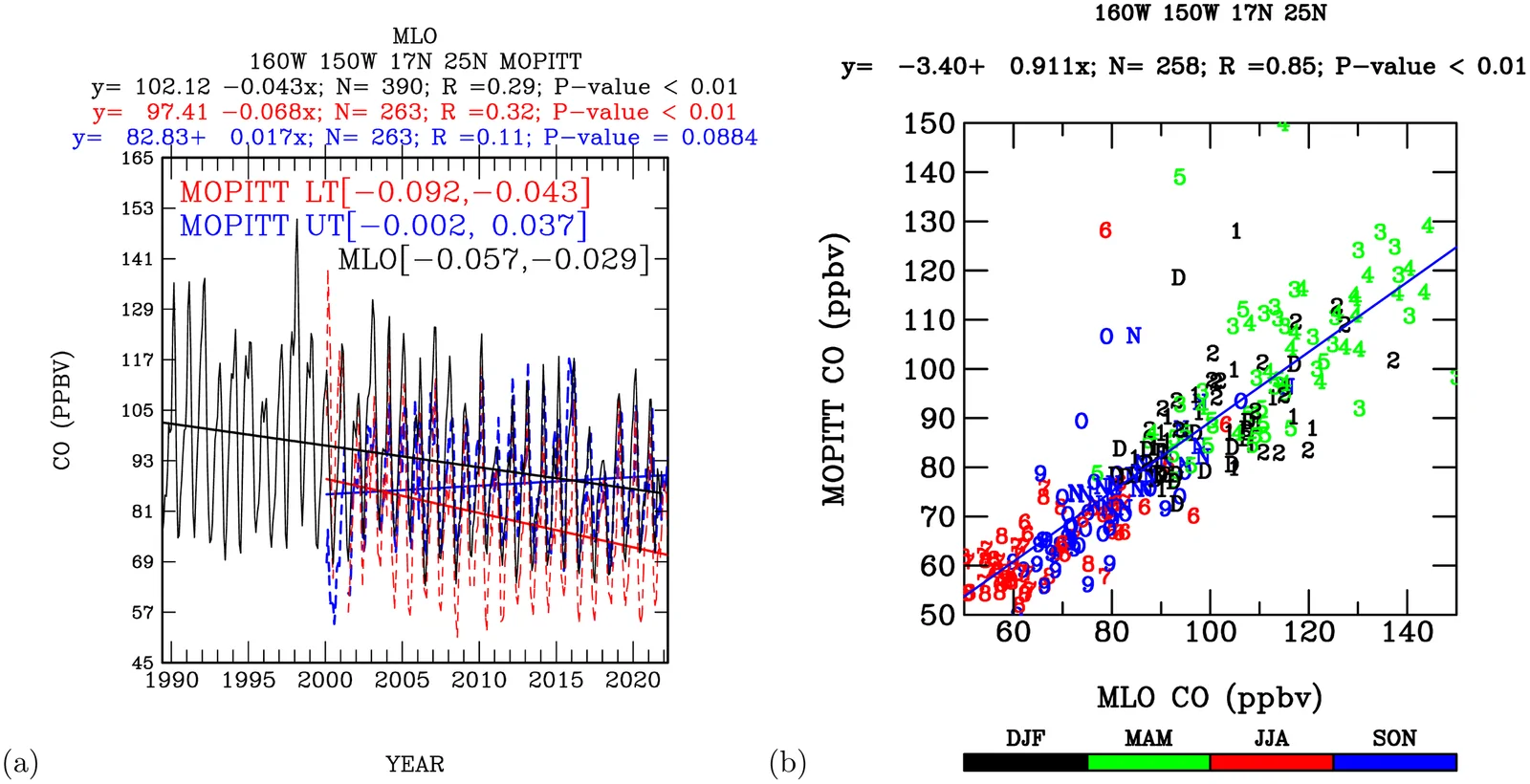
Time-series CO measurements from the MOPITT, and the comparisons with the Mauna Loa measurements. (a) The MOPITT CO measurements during the period 2000-2022 over the upper (blue) and the lower (red) troposphere in an area contains the Mauna Loa ($160^{\circ }W-150^{\circ }W,17^{\circ }N-25^{\circ }N$). The Mauna Loa measurements are shown for the period 1989-2022 (black). Also shown are the linear regressions (and 95% confidence intervals of the CO trends) of the MOPITT data over the lower and the upper troposphere, respectively; and the Mauna Loa data. (b) The MOPITT data over the lower troposphere correlated with the Mauna Loa measurements. The colors and the digits indicate the months of the data.

The MOPITT measurements are positively correlated with the Mauna Loa measurements (Fig 14(b)). However, the MOPITT overestimates CO during the summer to autumn months when the CO concentrations are lower than 100 ppbv, while underestimating CO during the winter to spring months when the CO concentrations are higher than 100 ppbv compared with the Mauna Loa measurements.

The linear regression exhibits negative trends in the CO from the Mauna Loa measurements during 1989–2022, which is consistent with the negative trends in the CO from the MOPITT measurements during 2000–2022 (Fig 14(a)). Conversely, the MOPITT measurements show positive linear regression over the Pacific upper troposphere, consistent with the IAGOS measurements (Fig 3).

For the lower troposphere over the Mauna Loa area, the Mauna Loa shows a decreasing CO trends with 95% confidence intervals of between −0.057 and −0.029, while the MOPITT data also shows a decreasing trends of CO with 95% confidence intervals between −0.092 and −0.043. For the upper troposphere, the MOPITT data shows an increasing CO trends with 95% confidence intervals between −0.002 and 0.037.

The long-term CO trend from Mauna Loa for the period 1989–2003 is −0.01 ppb *month*^–1^ with a 95% confidence interval of [−0.08, 0.05] (Fig 11(a)). When extending the analysis to 1989–2022, the Mauna Loa trend becomes more negative, at −0.04 ppb *month*^–1^ with a 95% confidence interval of [−0.06, −0.03] (Fig 14(a)). This enhanced negative trend reflects both the slowdown in anthropogenic CO emissions over Asia and reductions in emissions from other regions, including North America and Europe, particularly after 2004 (Fig 1(f)).

The MOPITT CO trends analyzed for the 2000–2022 period show a negative trend in the lower troposphere of −0.07 ppb *month*^–1^ with a 95% confidence interval of [−0.09, −0.04]. It is important to note that the MOPITT are more comparable with the Mauna Loa trends of the 1989–2022. The more negative Mauna Loa and the MOPITT trends over the full 1989–2022 period highlight the impact of emission reductions in recent decades.

Fig 15 compares MOPITT with IAGOS observations arranged following westerly flow patterns, starting from: (a) Downwind of the North America emission sources and over the North Atlantic ($50^{\circ }W$-$20^{\circ }W$, $10^{\circ }N$-$70^{\circ }N$). (b) Western Europe flight regions ($1^{\circ }E$-$15^{\circ }E$, $45^{\circ }N$-$55^{\circ }N$). (c) Paris region ($1^{\circ }E$-$10^{\circ }E$, $43^{\circ }N$-$53^{\circ }N$). (d) India ($73^{\circ }E$-$87^{\circ }E$, $10^{\circ }N$-$23^{\circ }N$). (e) China ($105^{\circ }E$-$125^{\circ }E$, $22^{\circ }N$-$45^{\circ }N$). (f) Downwind of the East Asia emission areas and over the North Pacific ($123^{\circ }E$-$142^{\circ }E$, $31^{\circ }N$-$42^{\circ }N$).

**Fig 15:**
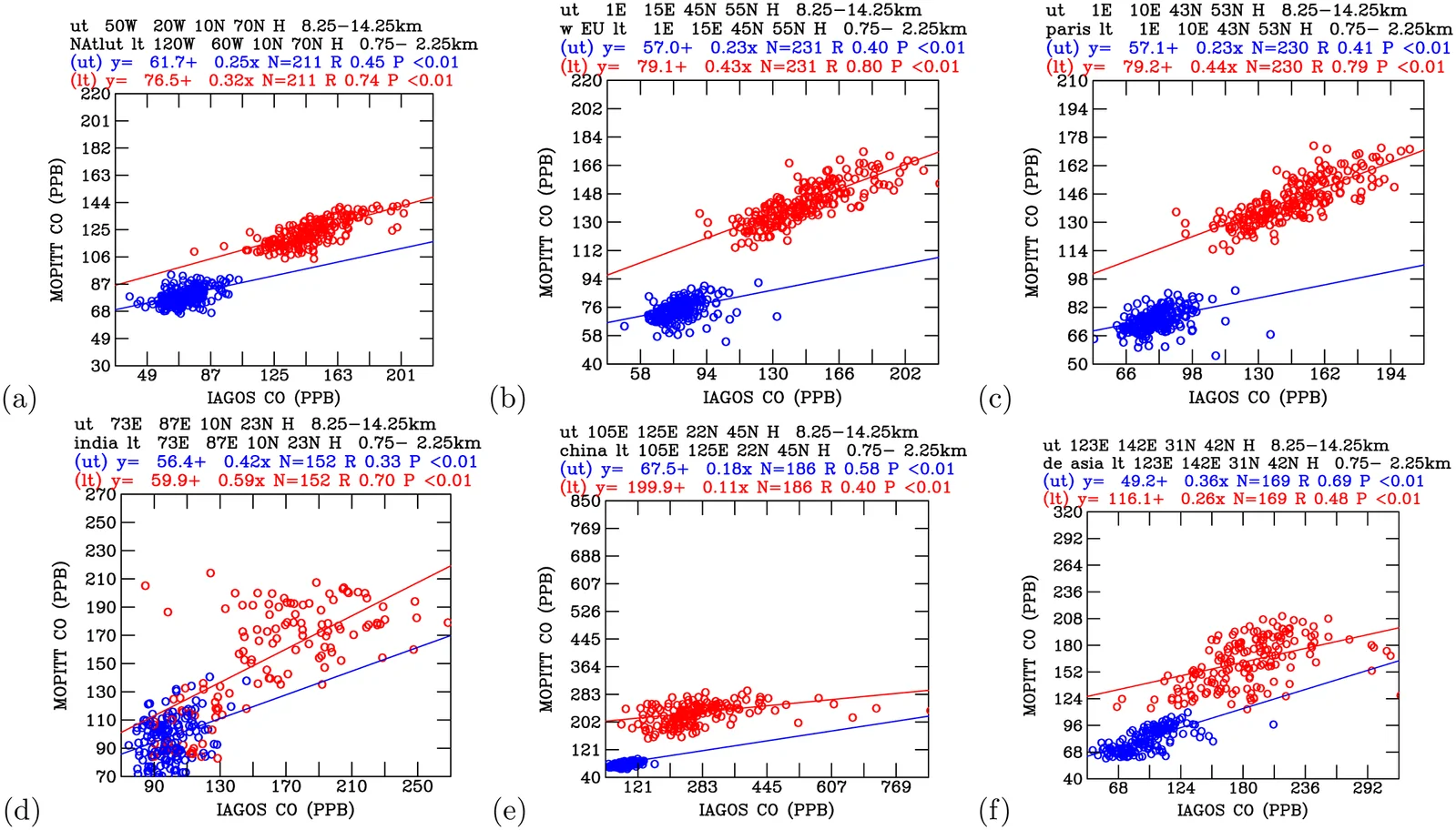
Comparisons of the MOPITT with the IAGOS observations. The panels are arranged following the westerly flow patterns, started from (a) downwind of the North America emission sources and over the North Atlantic ($50^{\circ }W-20^{\circ }W$, $10^{\circ }N-70^{\circ }N$), (b) western Europe flight regions ($1^{\circ }E-15^{\circ }E$, $45^{\circ }N-55^{\circ }N$), (c) Paris region ($1^{\circ }E-10^{\circ }E$, $43^{\circ }N-53^{\circ }N$), (d) India ($73^{\circ }E-87^{\circ }E$, $10^{\circ }N-23^{\circ }N$), (e) China ($105^{\circ }E-125^{\circ }E$, $22^{\circ }N-45^{\circ }N$), and (f) downwind of the east Asia emission areas and over the North Pacific ($123^{\circ }E-142^{\circ }E$, $31^{\circ }N-42^{\circ }N$). The measurements in the upper troposphere are shown in blue color, while the measurements in the lower troposphere are shown in red color.

Over the lower troposphere, MOPITT data correlated well but underestimated CO concentrations compared with the IAGOS measurements. Similarly, over the upper troposphere, MOPITT measurements also correlated well but underestimated CO concentrations compared with the IAGOS in-situ measurements. Hence, while the MOPITT data are well correlated with the IAGOS measurements, they tend to underestimate the CO concentrations compared with the IAGOS in-situ measurements.

The evolution of MOPITT versions is crucial for understanding biases. MOPITT V5, as per de Laat et al. [81], showed slight overestimation in the lower troposphere (less than 2 km) and potentially underestimation in the upper troposphere (5−9 km), though the paper focused more on NIR retrievals. MOPITT V6, as per Gaubert et al. [82], clearly underestimates in the upper troposphere by about 20%. More recent versions, such as V8 (released in 2019 and the version used in this work), have shown improvements in biases, as detailed in [51], which compared MOPITT V8 with NOAA aircraft data, finding biases ranging from −1.3% to 3.0% and drift less than 0.1% per year. Specific comparisons with IAGOS for altitude-dependent biases are less documented [51]. Given the improvements, it is likely that biases in V8 are reduced. However, the above results show that the MOPITT CO abundances generally underestimate the values from IAGOS.

Fig 16 presents time-series CO concentrations from the IMS 1984–2003 simulations and linear regression predictions for the period 2004–2022, compared with MOPITT and IAGOS observations in various regions of the upper troposphere.

**Fig 16:**
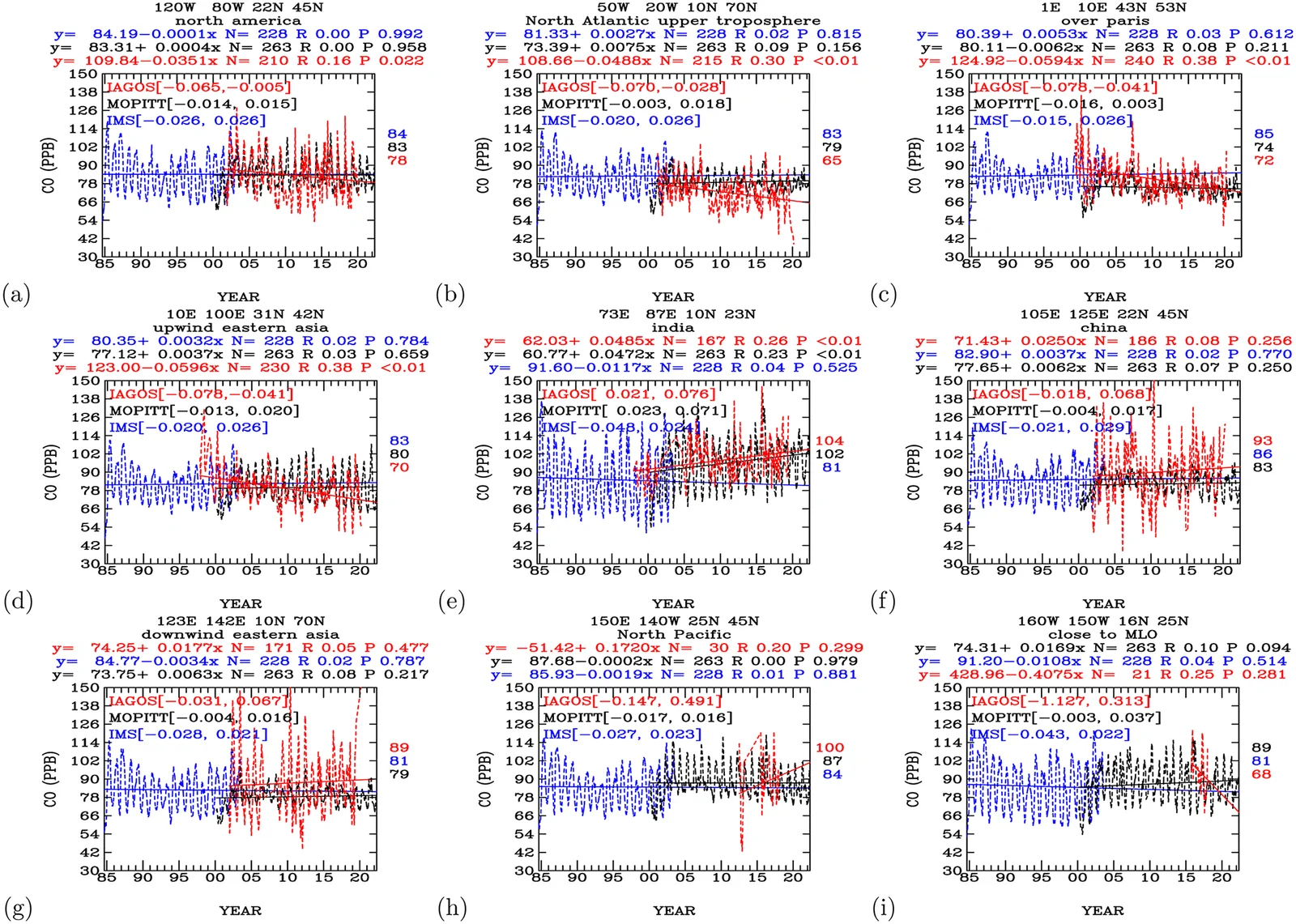
Time-series CO concentrations from IMS 1984-2003 simulations (blue), and linear regression prediction to the period 2004-2022 compared with the MOPITT (black) and the IAGOS observations (red) in the upper troposphere. The panels are arranged following the westerly flow patterns, started from over (a) the North America ($120^{\circ }W-80^{\circ }W$, $22^{\circ }N-45^{\circ }N$), (b) North Atlantic ($50^{\circ }W-20^{\circ }W$, $10^{\circ }N-70^{\circ }N$), (c) Paris region ($1^{\circ }E-10^{\circ }E$, $43^{\circ }N-53^{\circ }N$), (d) upwind of eastern Asia ($10^{\circ }E-100^{\circ }E$, $31^{\circ }N-42^{\circ }N$), (e) India ($73^{\circ }E-87^{\circ }E$, $10^{\circ }N-23^{\circ }N$), (f) China ($105^{\circ }E-125^{\circ }E$, $22^{\circ }N-45^{\circ }N$), (g) eastern Asian emission ($123^{\circ }E-142^{\circ }E$, $10^{\circ }N-70^{\circ }N$), (h) North Pacific ($150^{\circ }E-140^{\circ }W$, $25^{\circ }N-45^{\circ }N$), and (i) Mauna Loa ($160^{\circ }W-150^{\circ }W$, $16^{\circ }N-25^{\circ }N$). The 95% confidence intervals of the CO trends are shown on each figure.

The purpose is to verify the impact of continuous growth of CO emissions during the 2004–2022 period in the upper troposphere. The controlled and predicted CO trends from the constant emissions of CO and hydrocarbons are based on the IMS 1984–2003 simulations, which used constant emissions for each year from 2004 to 2022. The observed CO trends are derived from IAGOS in-situ measurements and MOPITT remote sensing measurements during the period 2004–2022, reflecting the impacts of real emissions of CO and hydrocarbons.

By comparing the observed CO trends with the controlled CO trends, we can identify the impacts of increased emissions of CO and hydrocarbons in the upper troposphere.

The comparisons are arranged following westerly flow patterns, starting from: (a) North America ($120^{\circ }W$-$80^{\circ }W$, $22^{\circ }N$-$45^{\circ }N$), (b) North Atlantic ($50^{\circ }W$-$20^{\circ }W$, $10^{\circ }N$-$70^{\circ }N$), (c) Paris region ($1^{\circ }E$-$10^{\circ }E$, $43^{\circ }N$-$53^{\circ }N$), (d) Upwind of eastern Asia ($10^{\circ }E$-$100^{\circ }E$, $31^{\circ }N$-$42^{\circ }N$), (e) India ($73^{\circ }E$-$87^{\circ }E$, $10^{\circ }N$-$23^{\circ }N$), (f) China ($105^{\circ }E$-$125^{\circ }E$, $22^{\circ }N$-$45^{\circ }N$), (g) Eastern Asian emission ($123^{\circ }E$-$142^{\circ }E$, $10^{\circ }N$-$70^{\circ }N$), (h) North Pacific ($150^{\circ }E$-$140^{\circ }W$, $25^{\circ }N$-$45^{\circ }N$), and (i) Mauna Loa ($160^{\circ }W$-$150^{\circ }W$, $16^{\circ }N$-$25^{\circ }N$).

The comparisons reveal that the IMS predicts CO trends in 2022 that are higher than the observed CO trends from IAGOS in-situ measurements and MOPITT remote sensing measurements over the upper troposphere of North America, the North Atlantic, Paris region, and upwind of the East Asia heavy emission areas.

For the remaining regions, IMS predictions are lower than the observations. IMS predictions are lower than both IAGOS and MOPITT measurements over India and the North Pacific. Over China and downwind of East Asia, IMS predictions are lower than IAGOS measurements and MOPITT observations are lower than IMS predictions over Mauna Loa. It is worth noting that there are few observations from IAGOS over the Mauna Loa area, and most available observations are actually higher than the trends predicted by the IMS model.

Hence, these results indicate that for regions upwind of the East Asia heavy emission area, the upper troposphere CO trends have decreased. Conversely, for the upper troposphere downwind of the East Asia heavy emission area, CO trends have increased during the period 2004–2022. We note that IAGOS trends are negative and significantly so for regions over the North America, North Atlantic, Paris region. Fig 16 shows that consistent CO trends are difficult to measure with the datasets mentioned in this work. There are variabilities as well regionally, depending on what datasets is considered. Continuous collection of in-situ measurements in the upper troposphere are needed to keep tracking CO trends over the North Pacific upper troposphere. For example, Figure Fig 16(h) and (i) are not very useful in terms of the IAGOS comparisons.

Fig 17 displays time-series CO concentrations from the IMS simulations (blue) and the linear regression prediction compared with MOPITT (black) and Mauna Loa observations (red). The negative CO trends from the IMS 1984–2003 simulations are consistent with the negative linear regression trends from the Mauna Loa in-situ measurements and the MOPITT remote sensing measurements.

**Fig 17:**
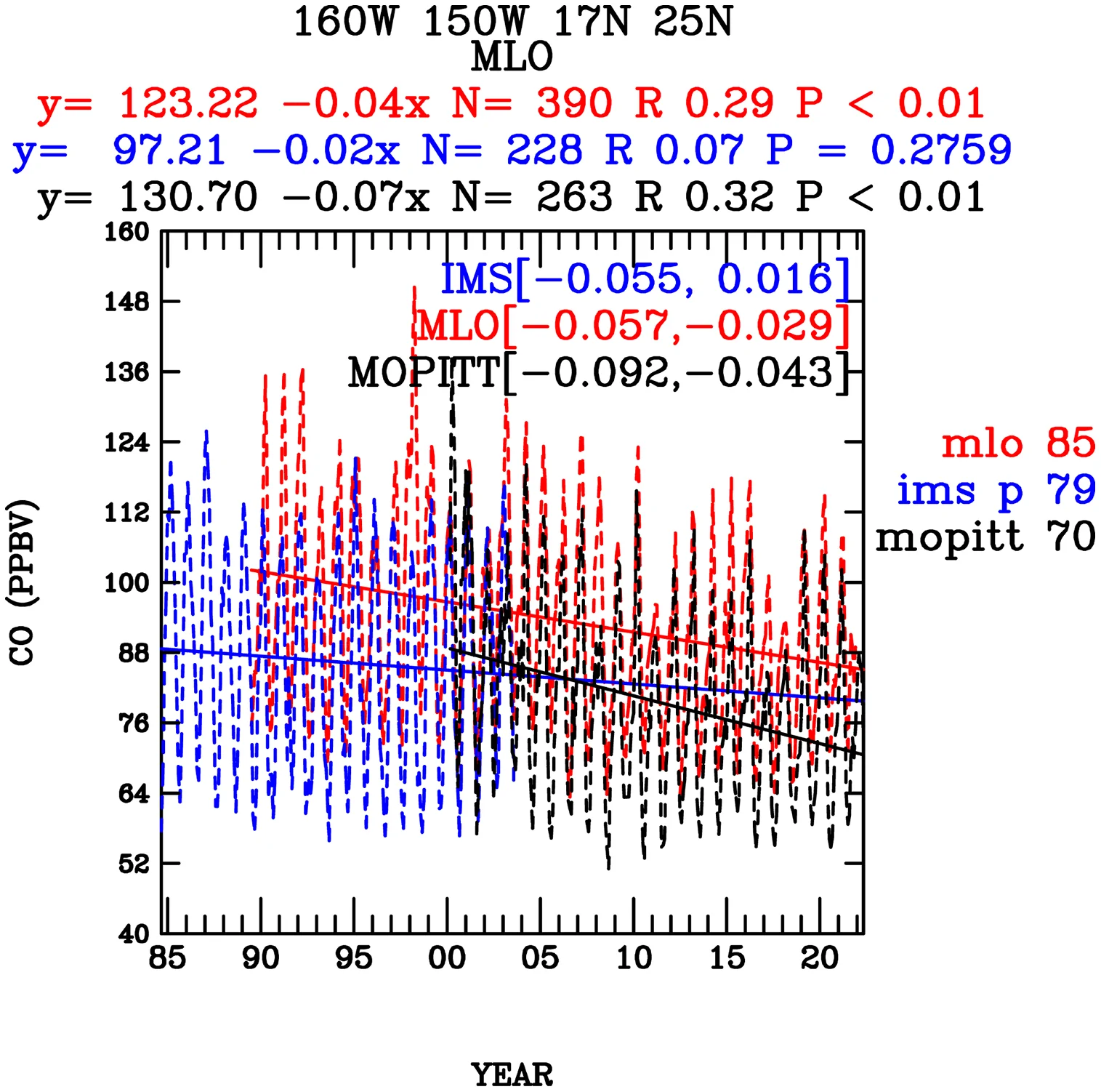
Time-series CO concentrations from IMS simulations (blue) and linear regression prediction compared with the MOPITT (black) and Mauna Loa observations (red) from the lower troposphere. Also shown are the 95% confidence intervals of the CO trends in brackets.

The CO concentration in 2022 from the IMS linear regression prediction (79 ppbv) is lower than the CO concentration from the linear regression prediction of the Mauna Loa 1989–2021 observations (85 ppbv) and higher than the CO concentration from the linear regression prediction of the MOPITT measurements (70 ppbv). The higher CO from Mauna Loa compared to the IMS predictions indicates that the actual annual CO and hydrocarbon emissions during 2004–2022 are higher than the constant emissions of CO and hydrocarbons used in the IMS 1984–2003 simulations.

The lower MOPITT prediction of CO compared to Mauna Loa observations is consistent with previous analyses (Fig 15(f)), which show that MOPITT measurements underestimate CO compared to IAGOS in-situ measurements in regions downwind of East Asia emission areas.

What controls long-term CO trends in the atmosphere? We can understand the processes governing CO dynamics through model simulations. The CO budget, calculated from a three-dimensional global tropospheric chemistry transport model IMS, indicates constant CO mixing ratios during the 30-year period of 1948–1978 (Fig 18(a)), and constant CO mixing ratios during the 20-year period of 1984–2003 (Fig 18(b)). The IMS model produce *O*_3_, which is controlled by the photochemistry in the model, compared well with long-term *O*_3_ measurements at Mauna Loa (Fig 18(c)). For the long-lived species such as *CO*_2_, the constant technological *CO*_2_ emissions does not match with the real-world *CO*_2_ emissions (Fig 18(d)).

**Fig 18:**
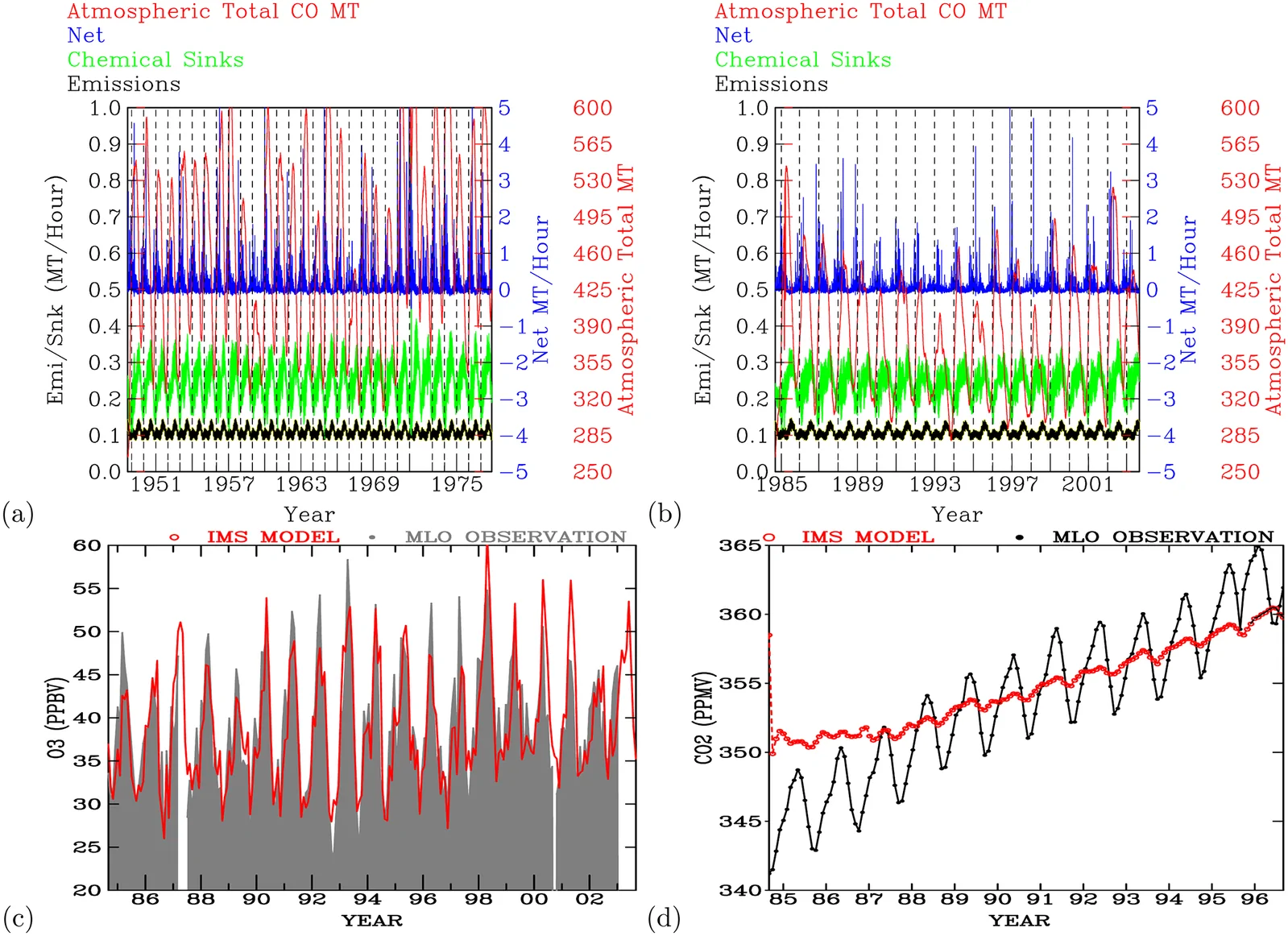
Time-series chemistry budget for CO concentrations from the IMS simulations. (a) The IMS 1948-1978 simulations. (b) The IMS 1984-2003 simulations. (c) The IMS 1984-2003 *O*_3_ simulations and comparison with Mauna Loa measurements. (d) The IMS 1984-2003 *CO*_2_ simulations and comparison with Mauna Loa measurements.

In these simulations, the total annual direct CO emissions are approximately 1100 MT per year [55]. These emissions consist of technological processes (set at constant emissions of 400 MT/yr), biomass burning emissions, and natural processes from the ocean and vegetation. Notably, EDGARv6.1 estimates total technological CO emissions at about 400 MT per year [83]. The IMS model calculates approximately 2200 MT of CO produced chemically (including direct CO emissions) per year, and approximately 2200 MT of CO removed chemically from the atmosphere per year. This indicates that the chemical sinks (2200 MT/yr) are roughly twice the CO emissions from direct sources (1100 MT/yr). The rates of chemical production (including direct CO emissions) of CO are balanced by the rates of chemical removal per year.

The total amount of CO in the atmosphere varies between 200 and 500 MT. According to EDGARv6.1 estimates, the total CO in the atmosphere is of the same order as CO directly emitted from technological processes. The total amounts of CO in the atmosphere fluctuate between 200 and 500 MT, depending on seasonal variations that drive chemical reactions converting CO to other products and converting other hydrocarbons to CO. Consequently, CO mixing ratios are predominantly influenced by chemical processes. The net chemical processes generally yield positive results from late autumn through winter to early spring months. Conversely, they tend to be negative from late spring through summer to early fall months. Consequently, the annual net chemical processes are close to zero, resulting in near-zero CO trends during the 20-year and 30-year model simulations, respectively.

The satellite remotely sensed CO trends during the period from March 2000 to May 2021 exhibit a range of behaviors, including near zero (Fig 16(a)) to negative trends (Fig 16(c)), and positive trends (as observed over India, Fig 16(e)). The global column amounts of CO trends derived from MOPITT observations during 2002–2018 are predominantly negative [49,50], indicating that, on a global scale, chemical sinks for CO exert a dominant influence on long-term mixing ratios in the atmosphere. Positive trends for CO are observed over areas such as India and downwind of East Asia emission sources over the North Pacific (Fig 16(f)), suggesting the influence of chemical sources in these regions. Overall, globally, CO mixing ratios are decreasing. Positive trends in CO are observed only in regions where chemical sources, arising from direct emissions of CO and hydrocarbons, outweigh chemical sinks, resulting in an increase in CO levels. Therefore, MOPITT provides valuable insights into CO trends on a global scale [26].

The monthly mean observations from Mauna Loa exhibit negative trends from 1989 to 2021 (Fig 17). While emission trends over Asian countries were positive during the 1978–2011 period, they turned negative during 2012–2018 (Fig 1). Consequently, the negative trends in monthly mean CO over Mauna Loa suggest that chemical sinks dominate over chemical sources in that region.

## Conclusions

In this work, we combine in-situ carbon monoxide (CO) measurements from the In-service Aircraft for a Global Observing System (IAGOS; 2012–2018) with earlier in-situ data from the NASA/NSF/NOAA experiments (1991–2019) to examine long-term trends of CO in the North Pacific upper troposphere. In addition, we utilize data from MOPITT satellite measurements and a global chemistry transport model to contextualize the trends observed in the IAGOS measurements. Our analysis reveals that the mean CO shows a positive trend of 0.15 ppb *month*^–1^ (95% CI: −0.02 to 0.32) or 18.0 ppb *decade*^–1^ in the region $150^{\circ }E-140^{\circ }W$ and $45^{\circ }N-60^{\circ }N$, and 0.06 ppb *month*^–1^ (95% CI: −0.04 to 0.16) or 7.2 ppb *decade*^–1^ in the region $150^{\circ }E-140^{\circ }W$ and $25^{\circ }N-45^{\circ }N$. Although these trends are not statistically significant at the 95% confidence level due to confidence intervals that include zero, the intervals are predominantly positive, suggesting that an increase in mean CO is more likely than a decrease. The monthly maximum CO exhibits a trend of 0.32 ppb *month*^–1^ (95% CI: −0.50 to 1.14) or 38.4 ppb *decade*^–1^ in the region $150^{\circ }E-140^{\circ }W$ and $45^{\circ }N-60^{\circ }N$, which is not statistically significant, and a statistically significant increase of 0.31 ppb *month*^–1^ (95% CI: 0.06 to 0.57) or 37.2 ppb *decade*^–1^ in the region $150^{\circ }E-140^{\circ }W$ and $25^{\circ }N-45^{\circ }N$. Conversely, the monthly minimum CO shows trends of −0.01 ppb *month*^–1^ (95% CI: −0.07 to 0.05) or −1.2 ppb *decade*^–1^ in the region $150^{\circ }E-140^{\circ }W$ and $45^{\circ }N-60^{\circ }N$, and −0.07 ppb *month*^–1^ (95% CI: −0.15 to 0.01) or −8.4 ppb *decade*^–1^ in the region $150^{\circ }E-140^{\circ }W$ and $25^{\circ }N-45^{\circ }N$, indicating a possible decrease, though not statistically significant as the intervals include zero. These findings suggest that over the period 1991–2018, the North Pacific upper troposphere has experienced an increase in the most polluted CO events, particularly in the mid-latitudes ($25^{\circ }N-45^{\circ }N$), where the monthly maximum trend is statistically significant. Meanwhile, background CO levels, represented by the monthly minimum, show a tendency to decrease, though not significantly. The overall mean CO may have increased, as indicated by the positive point estimates and predominantly positive confidence intervals, though these trends lack statistical significance at the 95% level. Our analyses show that the upper troposphere over the North Atlantic exhibits statistically significant decreasing CO trends. In contrast, the North Pacific upper troposphere shows trends suggestive of an increase in mean CO, with a statistically significant increase in monthly maximum CO in the mid-latitude region. These contrasting trends arise from specific regional differences rather than from generic regional variability: the North Pacific upper troposphere is ventilated by summertime convection and warm conveyor belts acting on Asian source regions whose emissions rose through most of the record, whereas the North Atlantic lies downwind of North American and European source regions whose emissions declined steadily and receives a larger fractional contribution of CO-poor stratospheric air.

We demonstrate that high-quality in-situ data from IAGOS are essential for analyzing trends in short-lived atmospheric species CO in the upper troposphere. Our IMS model simulations show that chemical sinks actively remove CO from the atmosphere, a process balanced by sources including direct emissions and the conversion of hydrocarbons to CO. Notably, the IAGOS data reveal some increasing trends over the North Pacific upper troposphere. In contrast, IAGOS shows some decreasing trends over the North Atlantic upper troposphere, highlighting the significant impacts of CO emissions control and reduction. By integrating IAGOS profile measurements with IMS chemical analysis, we discern that chemical sources dominate in the bottom half of the 2–10 km altitude range, whereas chemical sinks dominate in the top half of this range. Aircraft in-situ measurements reveal that the North Pacific upper troposphere experienced increased pollution over a 28-year period from 1991 to 2018. Elevated CO mixing ratios were more pronounced in summer than in winter, suggesting vertical transport of CO from surface sources. Model calculations corroborate the transport of surface CO from Asian sources as the primary contributor to increased pollution in the upper troposphere. Furthermore, these calculations support the notion that the rise in CO over the North Pacific upper troposphere is attributable to increased ground-level CO emissions.

In this work, we have drawn inferences about vertical profiles of chemical sources and sinks for CO near major anthropogenic source regions using in situ measurements from 2012 to 2018. We highlight regions over the North Pacific that show increasing CO trends in the upper troposphere, based on in situ aircraft observations from PEM-WEST A, PEM-WEST B, TRACE-P, HIPPO, IAGOS, and ATom. In contrast, regions over the North Atlantic, upstream of the Asian source areas, show decreasing CO trends. Furthermore, model simulations demonstrate that chemical processes play a key role in shaping CO profiles and trends, with pronounced sensitivity to variations in sources and sinks, as reflected in the analysis of CO percentiles (minimum, 25th, 50th, 75th, maximum) and mean values.
