# Supplementary material for: Long-term trends of carbon monoxide over the North Pacific upper troposphere from in-situ airplane measurements 1991–2018
Source: PLoS One. 2026 Oct 13;21(10):e0359855. doi: 10.1371/journal.pone.0359855 (PMC13660993; doi:10.1371/journal.pone.0359855)
Supplement: Supplementary file 1 [file pone.0359855.s002.pdf]

POINT-TO-POINT RESPONSE TO EDITORIAL OFFICE AND REVIEWERS

Manuscript Ref: PONE-D-25-57715 **Title:** Long-Term Trends of Carbon Monoxide Over the North Pacific Upper Troposphere From In-Situ Airplane Measurements 1991–2018 **Journal:** PLOS ONE

We thank the Academic Editor and both reviewers for their careful and constructive assessment. All comments have been addressed, and the changes are marked in the "Revised Manuscript with Track Changes" file. Line and section references below refer to the revised manuscript.

SECTION I: Responses to Editorial Office & Journal Requirements

[ED-1] Article splitting relative to doi:10.1371/journal.pone.0323460

Author Response:

We have provided an explicit clarification in both the Cover Letter and the Introduction of the revised manuscript regarding the scientific distinction between this study and our group's previous paper (Wang et al., 2025, PLOS ONE, 20(5): e0323460).

1. **Scope & objective.** Wang et al. (2025) was an initial characterization focusing strictly on an 8-month operational window (July 2012–February 2013) during the launch of the PGGM/IAGOS Pacific flight routes, capturing acute, short-term elevated CO pollution episodes (>160 ppbv) and back-trajectory attribution to ground sources.

2. **Multi-decadal synthesis.** The present manuscript is a 28-year long-term trend synthesis (1991–2018) integrating six airborne campaigns (PEM-West A/B, TRACE-P, HIPPO, ATom, IAGOS), MOPITT satellite observations, Mauna Loa ground baselines, and 3D CTM simulations (IMS).

3. **Novel methods & discoveries.** This manuscript applies a 10,000-sample perturbation regression framework to quantify trend rates and 95% confidence intervals across all statistical percentiles (pmin to pmax), uncovering a statistically significant increase in extreme polluted CO events (pmax) over the North Pacific mid-latitudes alongside decreasing background levels (pmin) — a percentile-stratified result that cannot be obtained from, and is not present in, the earlier 8-month episode study.

4. **No overlap of material.** No figure, table, or trend result in the present manuscript is reproduced from Wang et al. (2025). The 2012–2013 IAGOS data appear in the present work only as a small subset of a 28-year record, analysed for a different purpose.

This work is therefore a standalone multi-decadal climatological study rather than a split submission.

[ED-2] Please update your submission to use the PLOS LaTeX template

Author Response:

The manuscript has been reformatted using the official PLOS LaTeX template (http://journals.plos.org/plosone/s/lat
All text, equations, tables, and the bibliography now follow PLOS style: in-text citations are

numbered in order of appearance, and the reference list is formatted in Vancouver style. The resubmitted “Manuscript” (unmarked) and “Revised Manuscript with Track Changes” (marked-up) files compile cleanly under this template.

[ED-3] Code sharing guidelines

Author Response:

All code used in this work — the Fortran and Python analysis and data-processing routines that produce the trend statistics and figures, together with the Fortran source of the IMS chemistry transport model — is freely available, without charge and without restriction on use, to any interested researcher. The Code Availability section of the manuscript has been revised to state this explicitly.

The IMS model is a mature legacy Fortran code base, developed over more than two decades, whose formulation and evaluation are documented in three earlier peer-reviewed publications (Wang et al., 2001a, 2001b, 2001c) cited in the manuscript. Building and running it requires accompanying configuration files, input fields and usage instructions, and for this reason we supply it on request rather than as a static supplementary file, so that recipients receive a working configuration together with support in using it. Following the policy guidance that access should not rest on a single named individual, requests may be directed either to the corresponding author or to the Department of Atmospheric Sciences, National Central University, which will continue to provide access independently of any one author’s availability.

We confirm that no restriction of any kind — commercial, licensing, or otherwise — applies to the release of this code, and we are willing to deposit it in a public archive should the Editor prefer.

[ED-4] Grant numbers in ‘Funding Information’ and ‘Financial Disclosure’ do not match

Author Response:

We have audited and aligned all grant numbers across the online submission system and the cover letter. The award number NSC 97-2111-M-008-009, awarded to Kuo-Ying Wang by the National Science and Technology Council (NSTC), Taiwan, is uniformly declared.

[ED-5] Role of funder statement

Author Response:

We have included the exact required statement in our Cover Letter: “The funders had no role in study design, data collection and analysis, decision to publish, or preparation of the manuscript.”

[ED-6] Remove funding information from Acknowledgments

Author Response:

The Acknowledgments section has been cleaned of all references to funding bodies, grant numbers and ministries. It now exclusively thanks the participating airlines, the research consortia that provided data, and the reviewers.

[ED-7] Minimal data set

Author Response:

We confirm that our submission complies with the PLOS ONE Data Availability policy, and we have revised the Data Availability section accordingly.

1. **The data underlying the reported findings are already public.** Every CO measurement analysed in this study comes from a third-party archive that is permanently and publicly accessible without restriction: IAGOS, NASA GTE (PEM-West A/B, TRACE-P), HIPPO via NCAR EOL, ATom via ORNL DAAC, NOAA GML Mauna Loa, and MOPITT via NASA ASDC. Direct URLs and access dates are listed in the Data Availability section. Any researcher can obtain these data in exactly the same manner as the authors did, and can therefore reproduce the trend analyses reported here in full. The manuscript reports no measurement data that are not already openly archived.

2. **Derived quantities are reported in the article itself.** The trend slopes, 95% confidence intervals and P-values for every region, percentile and altitude layer are printed in the figures and tables rather than being held separately, so the quantities behind the stated conclusions are visible in the published record.

3. **IMS model output.** The raw output of the 30-year (1948–1978) and 20-year (1984–2003) IMS integrations is a legacy archive of substantial volume, produced over many years on now-superseded systems. It is offered openly and free of charge to any interested researcher, but its size makes deposition as a supplementary file impractical; it is therefore supplied on request. In line with the policy provision that access must not depend on a single named individual, we have named the Department of Atmospheric Sciences, National Central University as a durable institutional point of contact alongside the corresponding author, so that access is guaranteed independently of any one author's availability.

We would be glad to deposit a reduced subset of the model fields in a public repository if the Editor considers this necessary, and we welcome guidance from the Editorial Office on the preferred form.

[ED-8] Abstract must be identical in manuscript and submission form

Author Response:

The Abstract has been revised in this round (to define acronyms and to state the regional rationale, per Reviewer 1). We have updated the online submission form so that it matches the revised manuscript Abstract word for word.

[ED-9] Figure 1 may contain copyrighted map images

Author Response:

We confirm that all data used to produce Figure 1 are openly available, and that no copyrighted or proprietary map content is involved.

Figure 1 was not assembled from a downloaded map image. It was drawn by the authors using **NCAR Graphics**, the open-source scientific graphics package developed by the U.S. National Center for Atmospheric Research, which renders coastlines and political boundaries from its own built-in public-domain geographic database. The only other content in the figure is the flight-track

coordinates from the IAGOS/PGGM archive. No Google Maps, Google Earth, MapQuest or other copyrighted map service has been used, and no satellite imagery created using proprietary data appears in this or any other figure in the manuscript.

We note that figures produced with NCAR Graphics by this author group have been published under the CC BY 4.0 licence in PLOS ONE on two previous occasions, without any copyright difficulty arising:

- Wang K-Y, Nédélec P, Thouret V, Clark H, Wahner A, Petzold A. Elevated carbon monoxide observations over the North Pacific upper troposphere (July 2012–February 2013). PLoS One. 2025;20(5): e0323460.
- Wang K-Y, Nédélec P, Clark H, Harris N, Kajino M, Igarashi Y. Impacts on air dose rates after the Fukushima accident over the North Pacific from 19 March 2011 to 2 September 2015. PLoS One. 2022;17(8): e0272937.

No third-party permission is therefore required, and the figure is fully compatible with publication under CC BY 4.0. The Figure 1 caption has been updated to state that the map was generated by the authors using NCAR Graphics.

[ED-10] Upload new copies of Figures 2–16; detail is not clear

Author Response:

All due care has been taken to ensure that the figures are clearly readable.

We should explain the origin of the problem, since it is straightforwardly remediable. All figures in this manuscript were originally produced as **vector EPS** using NCAR Graphics. For the earlier version of this work the EPS files were converted to raster images in order to reduce the submission file size, and it is those reduced-resolution rasters that were supplied with the original submission. The loss of detail the Editor observed was introduced entirely by that conversion; it is not a limitation of the underlying figures.

We have therefore returned to the original vector EPS files and re-rendered every figure directly from them at publication resolution, bypassing the reduced rasters altogether. Because the source is vector, this produces genuine detail at any output resolution rather than interpolated pixels, and the resulting figures are correspondingly sharp. In preparing them we have confirmed that each figure falls within the specified pixel dimensions (789–2250 px wide, 2625 px maximum height) at a resolution within the 300–600 dpi range, is supplied as flattened RGB TIFF with LZW compression and no alpha channel, and is under the 10 MB file size limit. We have also reviewed each figure at final printed size to confirm that axis labels, legends and annotations are comfortably legible.

The original vector EPS files remain available, and we would be glad to supply any figure in EPS instead if the Editorial Office prefers that format.

SECTION II: Point-to-Point Response to Reviewer #1

We thank Reviewer 1 for the positive assessment of the writing and for identifying important recent literature that we had not previously considered.

[R1-1] Two important recent works on global CO variability and trends must be discussed

Author Response:

We thank the Reviewer for recommending these two studies, which we agree are directly relevant. Both have been integrated:

1. **Introduction.** A new paragraph discusses Joshi et al. (2023, *Chemosphere*, doi:10.1016/j.chemosphere.2023.139 who report a widespread global decline in CO attributed to the adoption of cleaner combustion technologies together with reduced fire activity in the burning hotspots, and Pathak et al. (2023, *Environmental Pollution*, doi:10.1016/j.envpol.2023.122269), who document 5–10% CO column reductions over regions of high anthropogenic activity during the COVID-19 lockdown but no comparable reduction where wildfires and agricultural waste burning dominate. We note explicitly that these studies establish a downward but strongly heterogeneous global tendency governed by the balance between emission controls and fire activity.

2. **Table 1.** Both studies have been added as rows to the summary table of long-term CO trend studies, with their data sources, periods and key findings, so that they can be compared directly against the other listed studies and against our own results.

3. **Reconciliation with our findings.** We state that our results are consistent with this global picture in the background air of the North Pacific upper troposphere, where the monthly minimum CO declines, while emphasising that the most polluted air masses (monthly maximum) over the mid-latitude North Pacific show the opposite tendency — a distinction that is visible only because we stratify the trend analysis by percentile.

[R1-2] Line 8: What is IAGOS?

Author Response:

The acronym is now spelled out on first use in the Abstract: “the In-service Aircraft for a Global Observing System (IAGOS)”. We have also spelled out MOPITT (Measurements of Pollution in the Troposphere) and IMS (Integrated Modelling System) on first use in the Abstract, as these were likewise undefined.

[R1-3] Line 10: Why this particular region? This has to be said in Abstract and in Introduction

Author Response:

An explicit rationale has been added to both locations. In the Abstract we now state that the North Pacific is the principal outflow corridor for Asian anthropogenic emissions — which have grown faster than those of any other region since the 1970s — yet is almost devoid of routine ground-based monitoring, so that its upper troposphere is simultaneously the atmosphere most exposed to the world’s fastest-growing combustion source and one of the least observed. In the Introduction, a dedicated paragraph develops this in three parts: the region’s role as the dominant export pathway for Asian pollution to the remote atmosphere and North America; the chronic observational gap (no long-term open-ocean surface sites, limited satellite vertical resolution in the

upper troposphere, and only short research-aircraft campaigns); and the consequent necessity of long-term in-situ measurements at cruise altitude.

[R1-4] Line 24: What is the reason for CO increase? This has to be written

Author Response:

We have added the physical explanation to both the Abstract and the Conclusions. The increase in the most polluted air masses reflects sustained growth in Asian combustion emissions over much of the record, combined with efficient summertime convective and warm-conveyor-belt lifting of boundary-layer air into the upper troposphere; because the upper troposphere is cold and dry, the correspondingly low OH abundances allow these CO enhancements to persist and be transported far downwind.

[R1-5] Lines 29–31: Please be specific rather than stating general reasons applicable to any region

Author Response:

The generic closing sentence has been replaced with region-specific mechanisms. The revised text states that the North Pacific upper troposphere lies immediately downwind of East and South Asian source regions whose CO emissions rose through most of the record, and is ventilated by strong summertime convection and warm conveyor belts associated with mid-latitude cyclogenesis; whereas over the North Atlantic, upwind North American and European emissions declined steadily throughout the period and a larger fractional contribution of CO-poor air descending from the lower stratosphere further dilutes upper-tropospheric CO. The opposing signs of the two trends therefore track the opposing trajectories of the upwind emission inventories, modulated by the transport pathway that dominates in each basin.

[R1-6] Line 124: CO is a greenhouse gas?

Author Response:

The Reviewer is correct, and we thank them for catching this imprecision. The text described CO as a “short-lived greenhouse gas”, which is not accurate, and we have corrected it.

The revised text states that CO is not itself a greenhouse gas in the radiative sense, but is a short-lived climate-relevant pollutant whose indirect forcing is nonetheless substantial. As the dominant sink of the hydroxyl radical, CO controls the oxidising capacity of the troposphere and thereby modulates the lifetime of methane and the photochemical production of tropospheric ozone, both of which are greenhouse gases; it is additionally oxidised to CO_2 itself.

We have also taken the opportunity to make the point quantitative rather than merely qualitative. Partitioning radiative forcing by emitted compound rather than by resulting concentration — as in Figure SPM.5 of the IPCC Fifth Assessment Report — CO emissions are assigned a radiative forcing of 0.23 [0.16 to 0.30] W m^{-2} over 1750–2011, delivered through the resulting changes in CO_2, CH_4 and O_3, with medium confidence. This exceeds the forcing attributed to N_2O (0.17 [0.13 to 0.21] W m^{-2}) and to the halocarbons (0.18 [0.01 to 0.35] W m^{-2}), and is roughly one seventh of that attributed to CO_2 emissions themselves (1.68 [1.33 to 2.03] W m^{-2}). We believe this both answers the Reviewer’s question precisely and strengthens the motivation for the long-term CO monitoring that is the subject of this manuscript: CO is not a greenhouse gas, yet on an emissions

basis its climate forcing is comparable to that of species routinely treated as major ones. The values are cited to IPCC (2013); the figure itself is not reproduced.

[R1-7] Lines 186–194: Compare the results with doi:10.1016/j.chemosphere.2023.139259

Author Response:

This comparison has been added, both as a Table 1 entry and in the surrounding discussion, contrasting the global 850 hPa decline reported by Joshi et al. (2023) with our percentile-stratified upper-tropospheric trends, and identifying the mechanism the two results share: the transition of East Asian emissions from growth to decline following the 2013 Clean Air Action, superimposed on continuing variability in fire activity.

[R1-8] Lines 375–382, 438–440: Compare your results with doi:10.1016/j.atmosenv.2022.119536

Author Response:

We thank the Reviewer for drawing our attention to this study, which we have now incorporated into the discussion of CO's chemical lifetime.

We should, however, be transparent about the nature of the connection. Kuttippurath et al. (2023) analyse a seasonal OH minimum over the **Indian Ocean**, whereas the present manuscript concerns the **North Pacific** upper troposphere. The two domains do not overlap, and no equivalent characterisation of the seasonal OH field is presently available for the North Pacific. A quantitative comparison of our CO measurements against their OH analysis would therefore not be meaningful, and we have not attempted one, since presenting a comparison across different ocean basins as though it were a like-for-like validation would mislead the reader.

What their result does provide is an important methodological caution, and this is how we have used it. Because OH is the dominant sink of CO, spatial and seasonal structure in OH translates directly into structure in the CO lifetime. Kuttippurath et al. demonstrate that basin-scale regions of depressed oxidising capacity exist and are seasonally organised — in their case most pronounced in spring with a secondary autumn minimum, arising from coupled SO_2–CO–O_3–H_2O chemistry. It follows that a single globally averaged CO lifetime is not an adequate basis for interpreting any regional CO record, including ours, and that where such a minimum is present CO enhancements survive further downwind than a uniform-OH assumption would predict.

We have therefore added this to the manuscript as a stated limitation and a direction for future work: establishing whether a comparable seasonal OH structure exists over the North Pacific would materially improve the attribution of the trends we report. We hope the Reviewer will agree that this is a more useful and more honest treatment of their recommended reference than a forced numerical comparison between two different oceans.

[R1-9] Lines 492–496: Discuss these recent CO trend studies here with other studies and your results

Author Response:

The discussion of long-term CO trends now synthesises our multi-decadal aircraft results with both 2023 studies, emphasising how Asian surface emission trends transition from positive before ~2012 to stabilising or declining after the 2013 Clean Air Action, and how this transition manifests differently in the different percentiles of upper-tropospheric CO: the background (pmin) follows the global

decline reported in those studies, while the polluted tail (pmax) over the mid-latitude North Pacific continues to increase.

[R1-10] Line 629: Lifetime of CO in the troposphere and its effect on Brewer–Dobson transport to the stratosphere

Author Response:

We have added a dedicated passage on this point. CO is removed almost entirely by reaction with OH, giving a tropospheric lifetime of roughly one to three months — closer to one month in the warm, humid, OH-rich tropical lower troposphere, extending to several months in the cold, dry upper troposphere and at high latitudes in winter, with a global average near two months. We then set out why this ordering of timescales matters. Convective lifting delivers boundary-layer air to the tropopause in a day or less, far faster than chemical loss, so the seasonal signature of surface emissions is preserved as air enters the lower stratosphere — the basis of the CO tape recorder. The subsequent ascent within the rising branch of the Brewer–Dobson circulation, however, takes several months to more than a year, which is long compared with the CO lifetime. The Brewer–Dobson circulation therefore transmits a strongly attenuated CO signal into the middle stratosphere: it controls the rate at which tropospheric air is supplied to the stratosphere, but most of the CO carried in that air is oxidised to CO_2 during ascent. A strengthening of the circulation consequently affects upper-tropospheric CO mainly through the compensating downward branch, which returns CO-poor stratospheric air to the extratropical upper troposphere, rather than by exporting appreciable CO upward.

SECTION III: Point-to-Point Response to Reviewer #2

We thank Reviewer 2 for a set of comments that has substantially improved the structure and transparency of the manuscript.

[R2-1] Temporal scope: extend analysis to at least 2023–2024 and update the title

Author Response:

We thank the Reviewer for this recommendation and have considered it carefully. After doing so we respectfully propose to retain the 1991–2018 boundary, for three reasons that we have now made explicit in the manuscript:

1. **Homogeneous multi-decadal epoch.** The 1991–2018 period forms a complete 28-year era that brackets the major policy transition in the dominant upwind source region, spanning both the rapid growth of East Asian emissions and the years immediately following China's 2013 Clean Air Action Plan. This allows the response of the upper troposphere to that transition to be examined within a single consistent record. It also matches the coverage of the EDGAR v6.1 gridded inventory (1970–2018) used throughout the model analysis, so that observed and emission trends are compared over identical periods.

2. **Statistical framework integrity.** Our trend significance rests on a 10,000-sample perturbation regression framework applied to sparse airborne records. Extending through 2024 would require changing MOPITT product version (V9 rather than the V8 TIR-only retrievals used here) and would introduce the pronounced, pandemic-driven discontinuity in commercial

flight density that affects IAGOS sampling from 2020 onward. Both changes would break the calibration and sampling homogeneity on which the present statistical treatment depends.

3. **Cross-instrument continuity.** Maintaining the 2018 cutoff preserves cross-instrument calibration continuity across all six platforms.

We have added this justification explicitly to the Data and Methods section, and we note there that post-2018 trends warrant a dedicated follow-up investigation using a sampling framework designed for the altered post-2020 flight matrix. We would of course be glad to reconsider if the Editor judges the extension essential; we note only that it would constitute a new study rather than a revision.

[R2-2] Introduction: strengthen rationale and state novel contributions

Author Response:

The Introduction has been revised to address all four elements:

- **Rationale:** the North Pacific is presented as the dominant conduit for trans-Pacific export of Asian pollution to the remote marine atmosphere and North America.
- **Monitoring gaps:** we emphasise the absence of long-term open-ocean surface sites, the limited vertical resolution of satellite column retrievals in the upper troposphere, and the few-week duration of dedicated research campaigns — making routine commercial-aircraft profiles indispensable.
- **Necessity:** we explain that long-term trend analysis of short-lived species such as CO is essential for validating CTM chemistry, OH budgets, and the atmospheric imprint of emission inventories on multi-decadal scales.
- **Novel contributions:** a new paragraph states these explicitly as (i) a 28-year multi-platform synthesis placing campaign-era and routine-monitoring-era observations on a common footing; (ii) a cross-platform consistency assessment quantifying how aircraft, satellite and model data differ in the same air masses; (iii) percentile-stratified trend detection separating background (pmin) from polluted (pmax) air; and (iv) regional attribution contrasting the mid-latitude and high-latitude North Pacific with the North Atlantic.

[R2-3] Datasets: clarify primary versus supplementary, give overviews and exact end dates

Author Response:

A new passage in the Data and Methods section classifies the datasets explicitly:

- **Primary:** IAGOS (in-situ backbone: routine calibrated high-frequency CO at cruise altitude plus ascent/descent profiles, constraining both absolute abundance and vertical structure); MOPITT (spatially continuous global context, used to place the regional trends within the global pattern and to test the representativeness of the aircraft sampling); the IMS chemistry transport model (separating chemical production and loss from transport); and the Mauna Loa flask record (independent continuously calibrated ground truth against which both model and satellite are evaluated).
- **Supplementary:** PEM-West A (1991), PEM-West B (1994) and TRACE-P (2001), which anchor the pre-IAGOS baselines and are used for distribution comparison rather than trend

fitting; and HIPPO (2009–2011) and ATom (2016–2018), used to check IAGOS values in overlapping air masses.

Exact coverage dates are now stated for each dataset, and Table 2 lists measurement methods, precision and accuracy uncertainties, and the combined root-sum-square errors.

[R2-4] Methods: decompose the core objective into defined subtasks and specify methods

Author Response:

We have added a new subsection, "Analysis Strategy and Statistical Methods", which decomposes the objective into the four subtasks the Reviewer identifies:

1. **Linear and perturbed trend detection.** Linear regression combined with a 10,000-sample Monte Carlo perturbation test in which each monthly value is perturbed within its ±7% measurement uncertainty, yielding distributions of slope, intercept and P-value rather than single values, so that reported significance reflects measurement error as well as scatter.
2. **Seasonal cycle evolution.** Monthly composites for the upper and lower troposphere examined by season (DJF, MAM, JJA, SON) and through upper–lower correlation within each season, isolating the periods in which vertical transport couples the two layers.
3. **Interannual variability and budget analysis.** Departures from the fitted trend examined against episodic drivers, with the IMS model (driven by NCEP reanalysis) used to separate dynamical variability from the effect of fixed versus evolving EDGAR emissions.
4. **Spatial and vertical heterogeneity.** Flight routes classified into regional domains (North Pacific, Euro-Asia, tropics, warm conveyor belt) and into vertical layers, so that trends are not aggregated across regions with different upwind sources and transport regimes.

We also make explicit the rationale for computing trends separately for the monthly maximum, the 75th, 50th and 25th percentiles, the minimum and the mean: the polluted tail and the background air respond to different drivers, and analysing the mean alone would conflate them.

We note the Reviewer's specific suggestion of the Theil–Sen estimator with a Mann–Kendall significance test, and STL decomposition for the seasonal cycle. We have considered these carefully and have retained the perturbed least-squares framework, for the following reasons.

The dominant uncertainty in these sparse airborne records is instrumental rather than distributional. Our framework propagates the ±7% measurement uncertainty of each individual observation into the reported slope distribution, which rank-based estimators such as Theil–Sen do not do: they are robust to outlying *values* but carry no information about the measurement error attached to each point. Given that the identification of genuinely elevated CO events is central to this manuscript, a method that treats extreme values as potential outliers to be down-weighted is also less well suited to our purpose than one that retains them and asks whether they are consistent with the stated instrumental uncertainty. Our approach additionally reports full confidence intervals rather than significance flags alone.

We agree that rank-based methods are valuable where residuals are strongly non-normal, and we would regard a Theil–Sen/Mann–Kendall treatment as a worthwhile independent cross-check in future work. We hope the Reviewer will accept that the present framework is appropriate to the data at hand and is fully documented in the revised Methods.

[R2-5] Results: unified metrics list and consistent subsection structure

Author Response:

The Results section now opens with a standardised metrics statement: trend slopes are reported in ppb month^{-1} and, for comparison with the wider literature, in ppb decade^{-1}; 95% confidence intervals are given as [CI_lower, CI_upper]; P-values from the 10,000-sample perturbation framework are reported; the Pearson correlation coefficient R is defined for each relationship examined; and, where model or satellite data are compared with in-situ measurements, the mean bias and correlation are given.

Regarding the Reviewer's mention of RMSE, we have adopted slope, confidence interval and P-value as the reporting standard rather than RMSE, because these address the question the manuscript actually asks. RMSE summarises how closely a fitted line reproduces the observations, which is a goodness-of-fit measure; our objective is instead to determine whether a trend is distinguishable from zero given the measurement uncertainty, which is precisely what the confidence interval and P-value quantify. This distinction matters here because the airborne records are sparse and episodically sampled, so a large RMSE may reflect genuine atmospheric variability — the pollution plumes that are themselves a central subject of this study — rather than a deficient fit, and would therefore be actively misleading as a quality metric. Our 10,000-sample perturbation framework goes further by propagating the $\pm 7\%$ instrumental uncertainty of each individual measurement into the reported slope distribution, so the stated significance reflects measurement error as well as scatter. We report the mean bias and correlation where satellite or model fields are compared against in-situ data, which is the appropriate agreement metric for those comparisons. We also state that each subsection follows the same order — analytical goal, data and method, key result with figure or table callout, and brief interpretation — and the subsections have been reorganised accordingly.
